# PGC-1α overexpression is not sufficient to mitigate cancer cachexia in either male or female mice

**DOI:** 10.1139/apnm-2022-0086

**Published:** 2022-06-14

**Authors:** Francielly Morena da Silva, Megan E. Rosa-Caldwell, Eleanor R. Schrems, Lauren Martinez, Madeline G. Amos, Seongkyun Lim, Ana Regina Cabrera, Jacob L. Brown, Tyrone A. Washington, Nicholas P. Greene

**Affiliations:** a Cachexia Research Laboratory, Exercise Science Research Center, Department of Health, Human Performance and Recreation, University of Arkansas, Fayetteville, AR, USA;; b Exercise Muscle Biology Laboratory, Exercise Science Research Center, Department of Health, Human Performance and Recreation, University of Arkansas, Fayetteville, AR, USA

**Keywords:** MitoTEMPO, mitochondria, *atrogin*, ubiquitin–proteasome, PGC-1*α*, cachexia, biological sex, Lewis lung carcinoma, muscle atrophy, MitoTEMPO, mitochondries, atrogine, ubiquitine-protéasome, PGC-1*α*, cachexie, sexe biologique, carcinome pulmonaire de Lewis, atrophie musculaire

## Abstract

Cancer cachexia (CC) accounts for 20%–40% of cancer-related deaths. Mitochondrial aberrations have been shown to precede muscle atrophy in different atrophy models, including cancer. Therefore, this study investigated potential protection from the cachectic phenotype through overexpression of peroxisome proliferator-activated receptor *γ* coactivator-1 α (PGC-1α). First, to establish potential of mitochondria-based approaches we showed that the mitochondrial antioxidant MitoTEMPO (MitoT) attenuates myotube atrophy induced by Lewis lung carcinoma (LLC) cell conditioned media. Next, cachexia was induced in muscle-specific PGC-1α overexpressing (MCK-PCG1α) or wildtype (WT) littermate mice by LLC implantation. MCK-PCG1α did not protect LLC-induced muscle mass loss. In plantaris, *Atrogin* mRNA content was 6.2-fold and ~11-fold greater in WT-LLC vs WT-phosphate-buffered saline (PBS) for males and females, respectively (*p* < 0.05). MitoTimer red:green ratio for male PGC was ~65% higher than WT groups (*p* < 0.05), with ~3-fold more red puncta in LLC than PBS (*p* < 0.05). Red:green ratio was 56% lower in females WT-LLC vs PGC-LLC (*p* < 0.05). In females, no change in red puncta was noted across conditions. *Lc3* mRNA content was ~73% and 2-fold higher in male and female LLC mice, respectively, vs PBS (*p* < 0.05). While MitoT could mitigate cancer-induced atrophy in vitro, PGC-1α overexpression was insufficient to protect muscle mass and mitochondrial health in vivo despite mitigation of cachexia-associated signaling pathways.

## Résumé

La cachexie cancéreuse représente 20 à 40 % des décès liés au cancer. Il a été démontré que les aberrations mitochondriales précèdent l’atrophie musculaire dans différents modèles d’atrophie, y compris le cancer. Par conséquent, cette étude a analysé la protection potentielle du phénotype cachectique par la surexpression de PGC-1α. Premièrement, pour établir le potentiel des approches basées sur les mitochondries, nous avons montré que l’antioxydant mitochondrial mitoTEMPO atténue l’atrophie des myotubes induite par les milieux conditionnés cellulaires du carcinome pulmonaire de Lewis (« LLC »). Ensuite, la cachexie a été induite par implantation de LLC chez des souris de même portée dans le muscle surexprimant de façon spécifique le PGC-1α (« MCK-PCG1α ») ou de type sauvage (« WT »). Le MCK-PCG1α n’a pas protégé la perte de masse musculaire induite par LLC. Dans le muscle plantaire, la teneur en ARNm d’*Atrogine* était 6,2 fois et 11 fois supérieure dans WT-LLC comparativement à WT-PBS pour les mâles et les femelles, respectivement (*p* < 0,05). Le ratio rouge/vert de MitoTimer pour les PGC mâles était d’environ 65 % supérieur à celui des groupes WT (*p* < 0,05) avec environ 3 fois plus de points lacrymaux rouges dans LLC que dans PBS (*p* < 0,05). Le ratio rouge/vert était d’environ 56 % inférieur chez les femelles WT-LLC comparativement à PGC-LLC (*p* < 0,05). Chez les femelles, aucun changement dans les points lacrymaux rouges n’a été noté dans toutes les conditions. La teneur en ARNm de *Lc3* était d’environ 73 % et 2 fois plus élevée chez les souris LLC mâles et femelles respectivement comparativement au PBS (*p* < 0, 05). Alors que MitoTEMPO pouvait atténuer l’atrophie induite par le cancer *in vitro*, la surexpression de PGC1α était insuffisante pour protéger la masse musculaire et la santé mitochondriale in vivo même en présence de l’atténuation des voies de signalisation associées à la cachexie. [Traduit par la Rédaction]

## Introduction

Cancer cachexia (CC) is a multifactorial syndrome occurring in up to 80% of cancer patients (Argilés et al. 2014). CC is characterized by increased catabolism in both skeletal muscle and adipose tissue, which can reduce a patient’s ability to respond to anticancer treatments (Ballarò et al. 2021) and accounts for 20%–40% of cancer-related deaths (Argilés et al. 2014; Tisdale 2002). Although CC management is crucial for patient survival, there are no currently available therapies capable of reversing or preventing it. Impaired energy metabolism in response to tumor-derived factors and an associated inflammatory response seems to largely influence muscle wasting in CC (Ballarò et al. 2021). Studies from our laboratory and others (Asp et al. 2010; Brown et al. 2017b; Ballarò et al. 2021; Lim et al. 2022) associate metabolic crisis due to mitochondrial impairments prior to the onset of CC and suggest a connection between mitochondrial aberrations and muscle atrophy preceding CC development. Therefore, promoting mitochondrial health and dynamics represents an interesting target for the development of therapies to prevent or mitigate CC outcomes.

Mitochondrial health is largely defined by respiratory function, reactive oxygen species (ROS) emission, and the quality of the interconnected network (regulated by control mechanisms including mitochondrial biogenesis, dynamics; fusion/fission and autophagy; mitophagy) (Yan et al. 2012; Russell et al. 2014; Rosa-Caldwell et al. 2020). Alterations in any of these mechanisms can negatively impact mitochondrial health and thus muscle health (Ballarò et al. 2021). Peroxisome proliferator-activated receptor *γ* coactivator-1 α (PGC-1α) is well known to promote mitochondrial biogenesis and has been demonstrated by our laboratory and others (Lira et al. 2013; Greene et al. 2015) to promote other aspects of mitochondrial quality control, including oxidative phosphorylation and ROS detoxification (Rius-Pérez et al. 2020). PGC-1α regulates the expression of several mitochondrial antioxidant genes, thereby playing a role in prevention of oxidative stress and resultant mitochondrial impairments (Valle et al. 2005; Vazquez et al. 2013). Furthermore, it has been demonstrated that dysregulation of PGC-1α induces detriments on redox homeostasis and exacerbates inflammation, promoting nuclear factor *κ*B activation (Rius-Pérez et al. 2020). Therefore, impaired mitochondrial health and quality control mechanisms are key derangements of CC and other atrophic conditions (White et al. 2012; Tzika et al. 2013; Puppa et al. 2014; Aversa et al. 2016; Brown et al. 2017b; Lee et al. 2020; Ballarò et al. 2021; Rosa-Caldwell et al. 2021), suggesting PGC-1α may be a compelling target to protect from cancer-induced muscle atrophies.

Mitochondrial aberrations are described not only in CC but also in other muscle wasting conditions (Brocca et al. 2010; White et al. 2012; Aversa et al. 2016; Brown et al. 2017b; Rosa-Caldwell et al. 2020). In fact, recent studies have explored the efficacy of targeting mitochondrial content as a therapeutic approach to various types of muscle atrophy such as hindlimb unloading-induced disuse atrophy and aging-induced muscle atrophy (Cannavino et al. 2014, 2015; Wang et al. 2017; Rosa-Caldwell et al. 2020; Ballarò et al. 2021). To date, studies targeting PGC-1α are equivocal, with some demonstrating PGC-1α overexpression is sufficient to protect muscle cross-sectional area or muscle mass with other models of atrophy previously mentioned, including disuse and denervation-induced atrophy (Sandri et al. 2006; Cannavino et al. 2014, 2015; Kang and Ji 2016; Wang et al. 2017), while others observed no protections with disuse and cancer-induced atrophy (Brault et al. 2010; Wang et al. 2012; Rosa-Caldwell et al. 2020). Although transgenic overexpression of PGC-1α to protect Lewis lung carcinoma (LLC)-induced atrophy has been explored previously, it is important to highlight significant differences in methodology, which could impact outcomes of this mitochondrial strategy against cachexia (Wang et al. 2012). Wang et al. utilized 4-month-old female mice only, with cachexia assessment accelerated and assessed at 2 weeks of tumor development, representing an earlier timepoint for cachexia compared with other LLC studies from the literature which commonly utilize endpoints around 4 weeks tumor development (Puppa et al. 2014; Brown et al. 2017b; H. Lee et al. 2017; Lim et al. 2022). Age of tumor inoculation, biological sexes, and duration of tumor development represent important scientific significance for cachexia phenotype. Changes in these variables could generate different outcomes in these studies. Moreover, our laboratory did not find sufficient evidence for protection of muscle cross-sectional area by transgenic PGC-1α overexpression during muscle disuse. Our prior findings do suggest PGC-1α overexpression blunted the ubiquitin–proteasome response to atrophy (Rosa-Caldwell et al. 2020) along with studies from other laboratories showing similar results in mitigated ubiquitin–proteasome induction in atrophy (Sandri et al. 2006; Brault et al. 2010; Cannavino et al. 2014, 2015; Kang and Ji 2016; Wang et al. 2017). Although different models of muscle atrophy display distinct mechanisms contributing to the development of muscle loss, the ubiquitin–proteasome system is a large contributor to CC-induced muscle wasting; this evidence suggests a potential protection from muscle catabolism by PGC-1α overexpression.

To our knowledge, studies investigating sufficiency of transgenic PGC-1α overexpression to protect from CC in LLC tumor-bearing mice are few (Wang et al. 2012). Likewise, most preclinical studies in CC are focused on males, not accounting for phenotypical disparities between biological sexes reported in multiple human cancer types, such as lung cancer (Cosper and Leinwand 2011). Previous studies from our laboratory indicate differences in biological sex in several distinct muscle atrophy models (Brown et al. 2017b, 2018; Lim et al. 2020, 2022; Rosa-Caldwell et al. 2021). In fact, males appear to be more so affected by inflammation-induced muscle atrophy models than females, such as cancer-induced atrophy, whereby females concomitantly display lower ubiquitin–proteasome activity when compared with males during CC (Kilgour et al. 2013; Brown et al. 2018; Vanderveen et al. 2018). Therefore, the purpose of this study was to investigate potential of mitochondria-directed approaches as strategy to alleviate cancer-induced atrophy. To accomplish this goal, we have taken a 2-fold approach: (1) in vitro antioxidant neutralization of mitochondrial ROS production by MitoTEMPO (MitoT), and (2) transgenic overexpression of PGC-1α in skeletal muscle of both male and female mice. To accomplish this dual purpose we first utilized LLC-conditioned media (LCM) as a strategy to recapitulate the cachectic effects of cancer in vitro (Zhang et al. 2011; Puppa et al. 2014; Brown et al. 2018), followed by LLC-induced cachexia in vivo with both wildtype (WT) and muscle-specific PGC-1α overexpressing (MCK-PGC-1α) mice. This study addresses biological sex differences in vivo, thus, adopts a novel study design when studying overexpression of PGC-1α in skeletal muscle and CC.

## Materials and methods

### Cell culture experiments

#### C2C12 culture

C2C12 myoblasts, reportedly derived from female specimens (Blau et al. 1983), were plated ~50000 cells per well in 6-well plates with 2 mL Dulbecco’s Modified Eagle Medium (DMEM) (Life Technologies, Carlsbad, CA, USA, 11965092) supplemented with 10% or 20% fetal bovine serum (Life Technologies, 26140079) and 1% Penicillin G and Streptomycin (pen/strep) (Life Technologies, 15140122) as per previous work by us (Lee et al. 2016; Brown et al. 2017a, 2018) and others (Call et al. 2017). Briefly, cell proliferation was allowed until ~70% confluence and differentiation was performed by switching media to DMEM supplemented with 2% horse serum, 1% pen/strep, 50% HEPES (Life Technologies, 15630080L), 0.75% transferrin (Life Technologies, 41400045), and 0.75% insulin (Novolin, 0169-1834-02) for 5 days, as previously described (Lee et al. 2016).

#### Lewis lung carcinoma-conditioned media treatment

LLC cells were grown up to 100% confluence as previously described, and media were collected and used as LCM (D.H. Lee et al. 2017; Brown et al. 2017b, 2018). LLC cells were incubated in DMEM supplemented with 10% fetal bovine serum and 1% pen/strep for 18 hours. The media were collected and filtered. LCM was diluted to a final volume of 25% in serum-free media. To account for dilution of conditioned media, and reduction of nutrient-deprivation effects, we utilized a control conditioned media (exposed to C2C12 for 18 hours to match LCM), adapted from prior works (Zhang et al. 2011; Puppa et al. 2014), as previously described (Jackman et al. 2017; Brown et al. 2018). For control groups, we utilized 25% of C2C12 growth media in serum-free media. C2C12 myotubes were treated with either control of LCM for 24 hours. All cell culture experiments were conducted in triplicate and repeated to ensure data accuracy.

#### MitoTEMPO treatment

To determine if neutralizing mitochondria-derived ROS may protect myotube diameter after LCM exposure, MitoT (SML0737, Sigma–Aldrich), a mitochondria-targeted antioxidant, was administered to C2C12 cells incubated in either control or LCM at a concentration of 2 μm. MitoT has a wide range of concentrations used in literature (1 nM–25 μm) (Eley et al. 2007; ; Russel et al. 2009; Lee et al. 2020).

#### Myotube diameter analysis

Myotube diameter analysis was performed as previously described (Lee et al. 2016; Brown et al. 2017a, 2018). Briefly, C2C12 myoblasts were allowed to proliferate until ~80% confluence and differentiated for 5 days. Myotubes were then divided into control or LCM, and appropriate vehicle or MitoT treatment. Myotubes were imaged using a 40× objective collecting 10 images per well, and measuring 3 to 4 myotubes per well. Five lines were drawn across each myotube’s diameter along the length of each myotube, and average was calculated as average diameter of each myotube (Brown et al. 2018). The same method was used for all the myotubes imaged. Researcher was blinded for this analysis and a second researcher repeated experiments with images acquired using a 40× objective and measurements as described earlier. These experiments were conducted in triplicate and repeated to test reproducibility and accuracy of the data.

#### MitoSOX

After 18 hours incubation in control or LCM with either vehicle or MitoT treatment, 5 μm MitoSOX Red (M36008, Invitrogen), a novel fluorogenic mitochondria-specific dye which on oxidation produces red fluorescence, in phosphate-buffered saline (PBS) was added to differentiated C2C12 cells for 10 minutes, rinsed, and cells were visualized at 510/580 nm (ex/em) on Nikon TiS epifluorescent microscope (Melville, NY) to assess mitochondrial superoxide production.

#### C2C12 myotube protein collection

Following LCM and MitoT treatment experiments, myotubes were collected for immunoblot by applying 100 μL of 2× protein sample buffer containing 0.23 M Tris–HCl, Ph 6.8, 4.5% *w/v* SDS, 45% glycerol, 0.04% *w/v* Bromophenol Blue, 80 mM dithiothreitol, 0.57 mM 2-mercaptoethanol, mini protease inhibitor cocktail (Roche, Indianapolis, IN, USA), and phosphatase inhibitor cocktails (Sigma–Aldrich, St. Louis, MO, USA). By using a cell culture scraper, cells were collected into 1.5 mL tubes with glass fiber filter, centrifuged, and denatured at 95 °C (Brown et al. 2018).

#### SUnSET protein synthesis

Protein synthesis of cell culture experiments was assessed by SUnSET protocol (Gwinn et al. 2008; Kobayashi 2015) as we have previously published (Lee et al. 2016; Brown et al. 2018). Briefly, 1 μm puromycin dihydrochloride (Calbiochem, Darmstadt, Germany) was added to cell culture media, followed by a 30-minute incubation prior to protein extraction. Immunoblotting protocol was followed as described above, using a 1:20000 dilution of mouse anti-puromycin IgG 2a antibody (EMD Millipore, Darmstadt, Germany; MABE343) followed by 1:20000 dilution of HRP-conjugated anti-mouse IgG fragment-specific secondary antibody (Jackson ImmunoResearch Labs, West Grove, PA). The entire lane was assessed for optical density, and this was then normalized to optical density of matching Ponceaus S stain.

#### Immunoblotting

Cells were collected as described above. Protein concentrations were measured using Reducing Agent and Detergent compatible protein Assay (RC/DC) assay (Bio-Rad, Hercules, CA, USA, 500–0119). A total of 40 μg of protein was loaded and ran on an SDS-PAGE gel, transferred to a PVDF membrane, and blocked in 5% milk in Tris-buffered saline (TBS). Membranes were probed overnight for primary antibodies specific to puromycin (Jackson ImmunoResearch, 115-035-206) and ubiquitin (Cell Signaling, 3933). Antibodies were diluted by using Odyssey Blocking Buffer TBS (LI-COR, 927-50000). LI-COR secondary antibodies conjugated with InfraRed were used according to the manufacturer’s protocol (LI-COR, 925-32211). Membranes were imaged on LI-COR Odyssey FC using IR detection. Entire lanes were normalized to the 45 kDa actin band of Ponceau S strain as a loading control. Ponceau S band densities did not differ between any groups. This was used was as a relative measure of the amount of actively translated proteins in the polysome prior to harvest of cells.

### Animal interventions

Animals with muscle-specific overexpression of MCK-PGC-1α were originally acquired from Jackson Laboratories (Stock: 008231, Bar Harbor, ME) and colony maintained in our laboratory (Rosa-Caldwell et al. 2020). Animals were kept on a 12:12-hour light–dark cycle and given access to normal rodent chow and water for the duration of the study in grouped cages. Mice (total *n* = 77) were then divided into 4 different groups within each biological sex: WT-PBS control, WT-LLC, MCK-PGC-1α (PGC)-PBS control, and PGC-LLC. All animal protocols were approved by the University of Arkansas Institutional Animal Care and Use Committee (AUP 17001).

#### Breeding/genotyping

At ~4 weeks of age, male and female mice were geno-typed for PGC-1α overexpression using genomic DNA isolated from the tail. MCK-PGC-1α was detected by end-point polymerase chain reaction (PCR) using the forward primer 5′-GCAGGATCACATAGGCAGGATGTGGCC-3′ and reverse primer 5′-GGAAGATCTGGGCAAAGAGGCTGGTCC-3′ with an initial denaturation at 96 °C for 4 minutes followed by 30 cycles of denaturation at 96 °C for 30 s, annealing at 60 °C for 30 seconds, extension at 72 °C for 45 seconds, and a final extension at 72 °C for 7 minutes. After genotyping, animals were randomly allocated in groups of *n* = 8–10 depending on colony and group as previously described in this paper.

### pMitoTimer electroporation and analysis

At 6 weeks of age, mice were electroporated with pMitoTimer, a tracer of mitochondrial quality. This was performed 2 weeks prior to cancer inoculation to avoid any confounding effects of procedure-induced damage on the final outcomes of this study. DH5-α *Escherichia coli* containing pMitoTimer plasmid were amplified and plasmid DNA isolated using PureLink HiPure Plasmid Filter Maxiprep kit (Life Technologies, K210017) (Brown et al. 2017b). For plasmid electroporation, briefly, animals were anesthetized with 2% isoflurane mixed with oxygen. Upon anesthesia, 10 μL of 0.36 mg/mL of hyaluronidase dissolved in sterile saline was injected into the right foot digitorum brevis (FDB) muscle of each mouse. After 1 hour of hyaluronidase injection (VWR, 10143-132), animals were re-anesthetized and the right FDBs were injected with 20 μg of pMitoTimer plasmid DNA (Addgene, Watertown, MA, 52659) diluted in 10 μL sterile saline. After 15 minutes of recovery, electroporation of FDB was performed, composed of 10 pulses at 75 V/cm, 1 Hz, and 20 ms/pulse. At tissue harvest, FDBs were removed and fixed in 4% paraformaldehyde:PBS solution for 20 minutes, then rinsed in PBS for 5 minutes. After washing in PBS, FDBs were carefully spread flat on gelatin-coated microscopice slides using forceps. FDBs were mounted with 50% glycerol:PBS solution and a standard microscope cover plate. pMitoTimer images were acquired at 40× magnification using the fluorescein isothiocyanate (FITC) (green, excitation/emission 488/518 nm) and tetramethylrhodamine (TRITC) (red, excitation/emission 543/572 nm) channels on a Nikon Ti-S inverted epifluorescent microscope (Melville, NY) with LED-based light source with controlled acquisition parameters to ensure no saturation of the signals and similar intensity of the green and red channels across samples. FITC and TRITC images were analyzed using Cell Profiler and MATLAB software (specialized coding, generous gift from Dr. Z. Yan) as we previously described (Brown et al. 2017b; Rosa-Caldwell et al. 2020, 2021). Images were quantified for red/green ratio and red puncta, measuring mitochondrial stress (greater red fluorescence relative to green fluorescence indicative of greater mitochondrial stress) and degenerated mitochondria (red fluorescent signal without any green, suggestive of completely degenerated mitochondria tagged for mitophagy). All samples were imaged the day they were collected and maintained in the dark to preserve fluorescence. Approximately 3 images were measured per animal with outcome variables averaged within each animal.

#### Lewis lung carcinoma allograft

LLC cells (ATCC, CRL-1642) were plated in 250 mL culture flasks in DMEM supplemented with 10% fetal bovine serum plus 1% penicillin and streptomycin. Once confluent, cells were trypsinized (Thermo Fisher, 25200072), counted, and diluted in PBS for implantation.

At 8 weeks of age, male and female mice were subcutaneously given an injection of either LLC cells (1 × 10^6^) suspended in 100 μL sterile PBS or equal volume of sterile PBS as control animals to the left hind flank. Tumors were allowed to develop for 4 weeks, a timepoint commonly associated with mild cachexia in this model (Brown et al. 2017b, 2018). Control mice were age-matched with 12-week-old mice.

### Grip strength

Beginning at 8 weeks of age, mouse grip strength was tested weekly by using a rodent grip strength meter (Harvard Apparatus, Holliston, MA, USA). Briefly, each mouse was allowed to hold on to a metal grid connected to a force transducer and then gently pulled backward in the horizontal plane by the tail off the grasping grid. Each mouse was submitted to 3 sets of 5 consecutive repetitions with a 2–3 minutes of rest period between each set, then the mean was taken to determine grip strength for each mouse as previously described (Smith and Tisdale 1993). To account for differences in body size across mice, grip strength data were normalized to body weight.

### Tissue collection

Tissue collection was performed at 12 weeks of age, following the 4 weeks (± 2 days) of tumor growth. Mice were anesthetized with isoflurane prior to euthanasia and tissue wet weight of gastrocnemius, plantaris, soleus, extensor digitorum longus, tibialis anterior (TA) muscles of both limbs, along with heart, spleen, liver, and gonadal fat was assessed. All tissue and blood samples were snap-frozen in liquid nitrogen and stored at −80 °C for further utilization.

### mRNA analysis

#### Total RNA isolation, cDNA synthesis, and RT-qPCR

Plantaris and TA muscles were used for reverse transcription quantitative polymerase chain reaction (RT-qPCR) analyses. Total RNA was extracted using TRIzol reagent (Life Technologies, Grand Island, NY). Following phenol–chloroform extraction, RNA was ethanol precipitated and diluted in 75% of diethyl pyrocarbonate-treated ethanol. RNeasy kit (Ambion kit) was used to further isolate total RNA. Total RNA was dissolved in water, concentration and purity were determined using a BioTek Take3 micro-volume microplate with a BioTek PowerWave XS microplate reader (BioTek Instruments Inc., Winooski, VT), 260/280 nm ratios and RNA concentrations were obtained. Samples were only used if 260/280 ratios were of acceptable (>2.0) quality. Samples were stored at −80 °C until further use. RNA was reverse transcribed to cDNA from 1 μg of total RNA using Superscript Vilo cDNA synthesis kit (Life Technologies) in a final volume of 20 μL at 25 °C for 10 minutes, followed by 42 °C for 50 minutes and 70 °C for 15 minutes. PCR was performed using QuantStudio 3 Real-Time PCR system (Applied Biosystems). A 25 μL reaction composed by adequate amount of TaqMan probes or SYBR Green primer pairs, plus TaqMan Universal Master Mix or Power SYBR Green Master Mix (Applied Biosystems) was used to amplify cDNA. Samples followed a protocol consisting of incubation at 95 °C for 4 minutes, followed by 45 cycles of denaturation, annealing, and extension at 95 °C and 60 °C. TaqMan and SYBR Green Fluorescence were measured at the end of the extension step of each cycle. Fluorescence-labeled Taqman probes included: *18S* (Clone 203 #Mm03928990_g1), *Pgc1*α*1* (Clone #Mm01208835_m1), *Lc3* (Clone #Mm00458725_g1), *Redd1204* (Clone #Mm00512504_g1), *Deptor* (Clone #Mm01195339_m1), *Atrogin* (Clone #Mm00499523_m1), *MuRF1* (Clone #Mm01185221_m1), *p62* (Clone #Mm00448091_m1), *Ubiquitin C* (UBC) (Clone #Mm02525934_g1), *Gadd45a* (Clone #Mm00432802_m1), *Atp2a1* (*Serca1*) (Clone #Mm0125320_m1), and Ryr1 (Clone #Mm01175211_m1) or SYBR primers for *Bnip3*, *Opa1*, *Beclin* as described previously (Greene et al. 2015) along with *Pgc1α4* as follows: Forward: TCACACCAAACCCACAGAAA, Reverse: CTGGAAGATATGGCACAT. PCR was run for 50 cycles at a melting temperature of 54 °C for the *Pgc1α4* primer pair. All probes and primers were purchased from Applied Biosystems. Results were analyzed using QuantStudio Software. Cycle threshold (*C*_T_) was determined, and the Δ*C*_T_ value was calculated as the difference between *C*_T_ value and 18S *C*_T_ value. 18S *C*_T_ values were not different between experimental conditions. Final quantification of gene expression was calculated using the ΔΔ*C*_T_ method. Relative quantification was calculated as $2^{-\Delta \Delta C_{\text{T}}}$.

### Statistical analysis

For cell culture, independent factors included media (Con or LCM) and drug treatment (Vehicle or MitoT) Control (Con) Vehicle, LCM Vehicle, Con MitoT, LCM MitoT. Data were analyzed by 2-way analysis of variance (ANOVA) with factors of media (Con vs LCM) and pharmacological inhibition (Vehicle vs MitoT). Here significant *F* ratios were found and Student–Newman–Keuls post hoc test was used to delineate differences among means. For all experiments, the comparison-wise error rate, α (P), was set at 0.05 for all statistical tests. All data were analyzed and graphed using GraphPad Prism (La Jolla, CA, USA) and expressed as mean ± SEM.

Results of animal-associated experiments are reported as mean ± SEM. Transgenic colony and sex were analyzed by paired 2-way ANOVA. Independent factors within sex included intervention group (LLC or WT control) and genotype (WT or PGC). Biological sex was not included as a statistical factor because the primary purpose of this study was to evaluate the influence of PGC on muscle health during CC, not necessarily make statistical comparisons of sex. However, it was important to include both males and female mice in this study. Additionally, a 2 × 2 × 2 ANOVA would inflate number of comparisons and result in an overall underpowered study (Rosa-Caldwell et al. 2020). When a significant interaction *F* ratio was found within sex, pairwise comparisons between groups were completed by using Tukey’s post hoc adjustment. Statistical significance was determined if α (P) ≤ 0.05. Additionally, an unpaired *t* test analysis for baseline comparisons of male WT-PBS vs female WT-PBS was performed for all qPCR targets, with statistical significance determined if α (P) ≤ 0.05.

## Results

### Treatment with MitoTEMPO in LCM-treated C2C12 is sufficient to mitigate myotube atrophy and oxidative stress

**Fig. 1.**
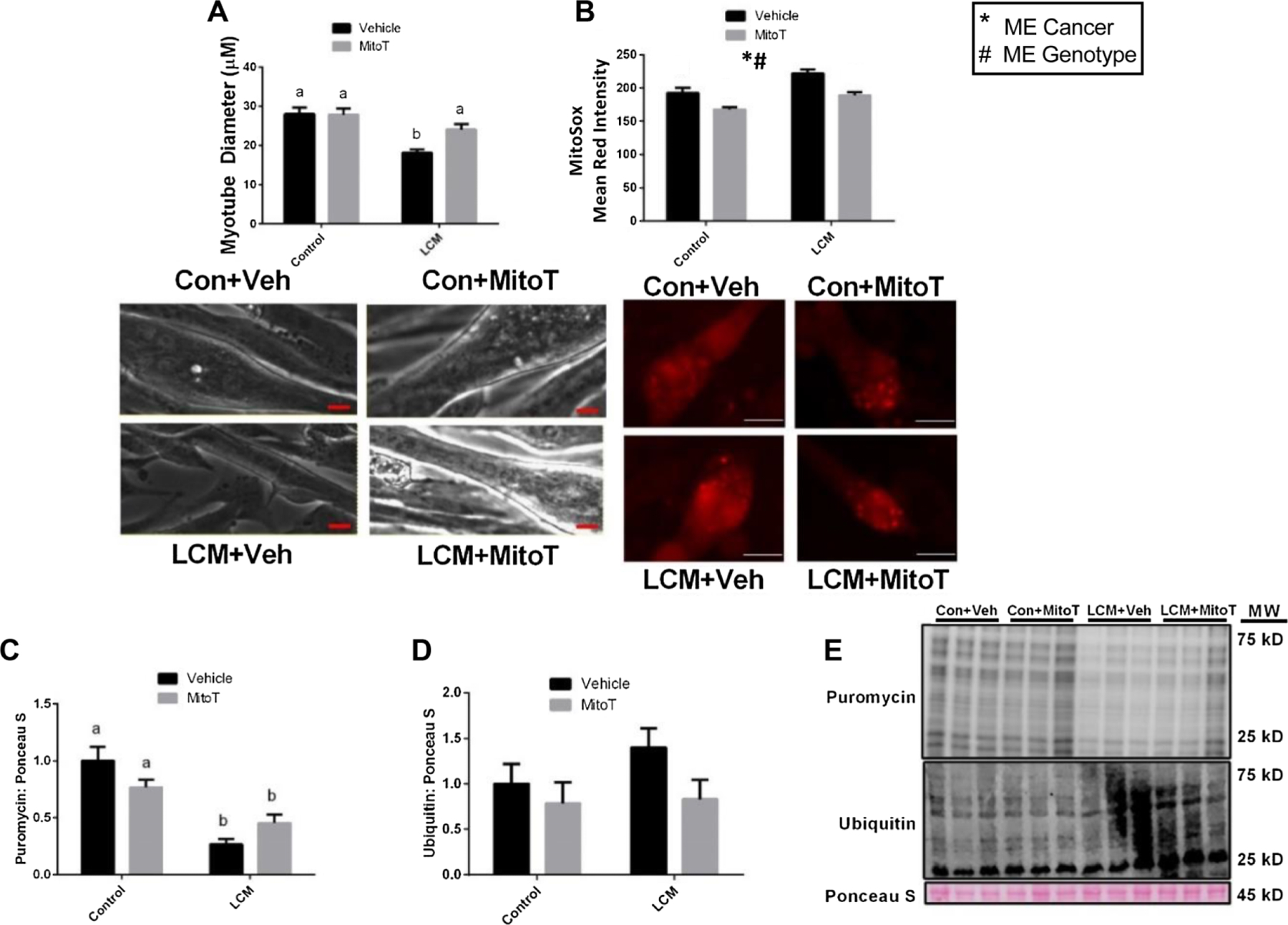
MitoT protects against LCM-mediated loss of myotube diameter. (*a*) Myotube diameter analysis of control media + vehicle, control media + MitoT, LCM + vehicle and LCM + MitoT. (*b*) MitoSOX analysis of control media + vehicle, control media + MitoT, LCM + vehicle, and LCM + MitoT. (*c*) Puromycin incorporated for groups control media + vehicle, control media + MitoT, LCM + vehicle, and LCM + MitoT after 30 minutes puromycin treatment following 18 hours of treatment. Scale bars: 20 *μ*m. (*d*) Protein content of ubiquitin following 18 hours of control media + vehicle, control media + MitoT, LCM + vehicle, or LCM + MitoT treatment. All measured in C2C12 myotubes and normalized to and Ponceau S. (*e*) Representative immunoblot images for each protein of interest taken in order from same membrane. Data shown in mean ± SEM. Different lowercase letters denote an interaction effect considering Tukey’s adjusted *p* < 0.05. An *n* of 6 replicated (wells) per condition was used in 2 independent experiments. Main effect for cancer (CON vs LLM) is denoted by *. Main effect for genotype (Veh vs MitoT) is denoted by #, *p* <0.05.

To investigate the potential of mitochondria-targeted strategies against cancer-induced atrophy, we performed cell culture experiments with C2C12 exposed to LCM + Vehicle (LCM + Veh) or LCM + MitoTEMPO (LCM + MitoT) with matching controls. Myotube diameter of C2C12 LCM + Veh was ~40% smaller than both control groups (*p* < 0.05, Fig. 1a), while LCM + MitoT was not statistically different from Control (Fig. 1a). Accordingly, MitoSOX fluorescence from C2C12 myotubes (indicative of oxidative stress) was higher in LCM + Veh by ~10% when compared with both Con + Veh and LCM + MitoT groups, and 30% higher when compared with Con + MitoT group (*p* < 0.05, Fig. 1b), while LCM + MitoT was not different from Con-Veh. Lastly, to assess protein synthesis and protein degradation, we performed the SUnSET assay and immunoblotted for protein ubiquitination. Puromycin protein levels were ~50% lower in both LCM groups when compared with control groups (*p* < 0.05, Figs. 1c and 1e), while ubiquitin protein levels did not reach statistical significance (Figs. 1d and 1e). These data suggested that mitochondria-targeted approaches may protect from cancer-induced muscle wasting.

### Confirmation of transgenic models and cachectic state

**Fig. 2.**
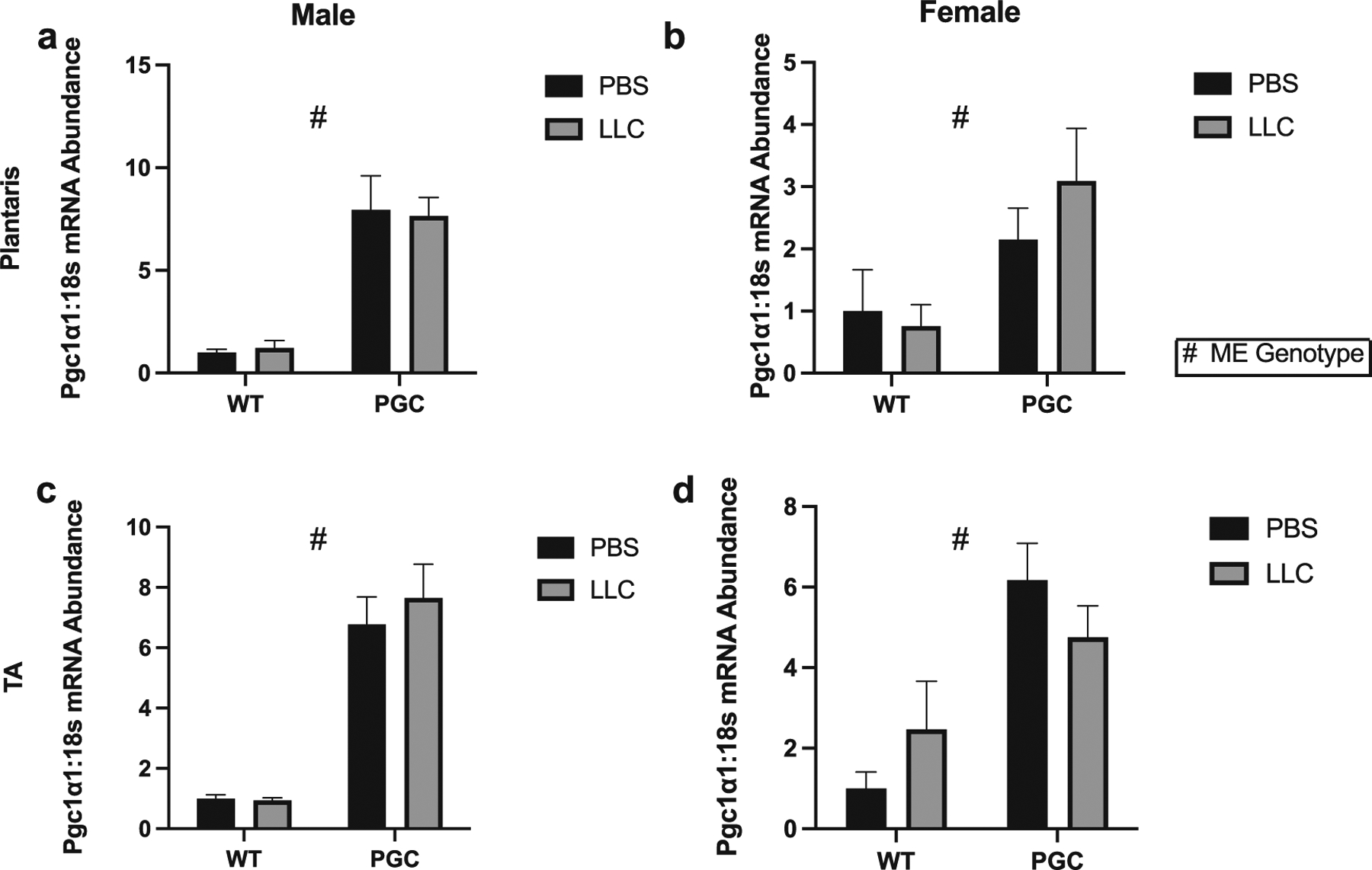
PGC-1α overexpression genotype confirmation. mRNA content of PGC-1α in both plantaris and tibialis anterior (TA) of males (*a*, *c*) and females (*b*, *d*). Data are shown in mean ± SEM. Main effect for genotype (WT vs PGC-1α) is denoted by #, *p* < 0.05. An *n* of 5–10 animals per group was used. Individual data point graphs for RT-PCR targets are available in Supplementary Fig. S2.

Considering MitoT was able to protect from cancer-induced myotube atrophy in vitro and recent data suggesting the mitochondria-targeted compound SS31 may have protective effects in this condition (Ballarò et al. 2021). We next utilized PGC-1α muscle transgenic mice to assess the efficacy of general targeting of mitochondrial quality control mechanisms to protect from CC, since PGC-1α can coordinate the expression of several antioxidant genes to protect cells against oxidative stress damage (Greene et al. 2015; Deng et al. 2020; Rius-Pérez et al. 2020). MCK-PCG1α transgenic mice had 8- and 2-fold greater *Pgc1α* mRNA content compared with WT in male and female mice similar to our prior reports (*p* < 0.05, Fig. 2) (Greene et al. 2015; Rosa-Caldwell et al. 2017, 2020). Baseline expression of *Pgc1α* was not different on male-WT-PBS vs female-WT-PBS (Supplementary Fig. S1a and S1b). Body weights were not significantly different between groups after the 4 weeks of tumor development in either male or female mice; however, body weight:tumor weight ratios were ~10% lower in LLC groups compared with PBS control in females, but not male mice (*p* < 0.05, Tables 1a and 1b). In addition, we observed lower wet muscle weight of soleus mass (~24% lower) and plantaris mass ~15% lower in WT-LLC vs PGC-PBS in females, gastrocnemius mass was ~12% lower in LLC vs PBS groups (*p* < 0.05, Table 1a). In females, TA mass was ~10% lower in LLC group vs PBS (*p* < 0.05, Table 1a). In males, ~15% lower TA mass in LLC vs PBS (*p* < 0.05, Table 1b). Furthermore, gonadal fat mass in females was 52% lower in LLC compared with PBS group (*p* < 0.05, Table 1a). In females we found a ~3- and 7-fold greater spleen mass in WT-LLC and PGC-LLC groups respectively, compared to PBS (*p* < 0.05, Table 1a). In males, gonadal fat mass was lower ~42% in LLC groups when compared with PBS along with an interaction in spleen mass (*p* < 0.05, Table 1b). In males, spleen mass showed an interaction with WT-LLC being ~4-fold higher than both WT-PBS and PGC-PBS, and 58% higher spleen mass than PGC-LLC, while PGC-LLC was 2.6-fold higher spleen mass than both WT-PBS and PGC-PBS (*p* < 0.05, Table 1b).

### Pgc1α overexpression exhibits minimal impacts on voluntary force production and calcium regulatory genes

**Fig. 3.**
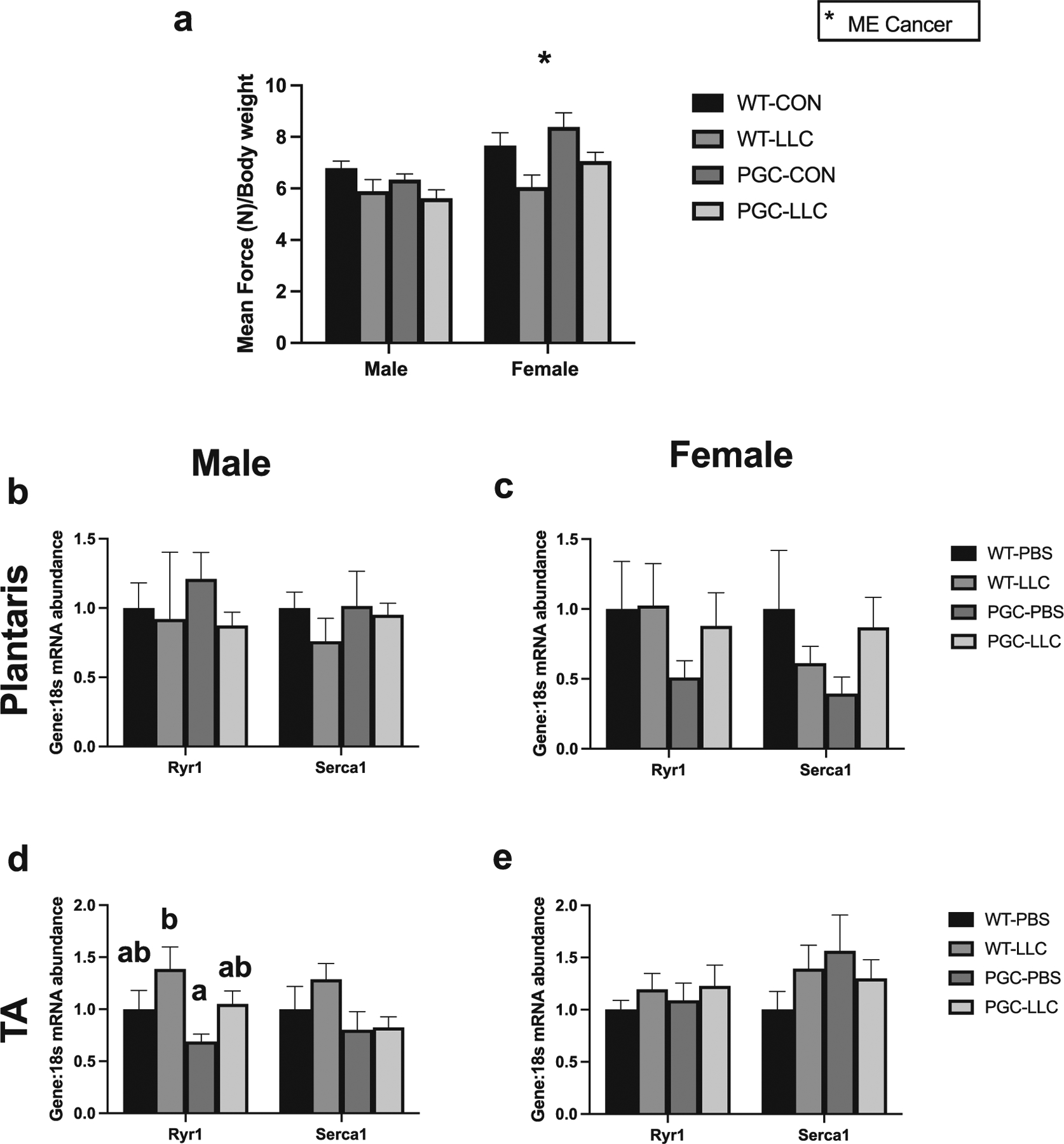
LLC affects mean force in females but not males with no apparent changes in calcium modulation-associated genes at the mRNA level. (*a*) Mean force (*g*) normalized by BW on time course measures of grip strength in male and females. mRNA content of *Ryr1* and *Serca1* in both plantaris and tibialis anterior (TA) of males (*b*, *d*) and females (*c*, *e*). Data are shown in mean ± SEM. Main effect for cancer (CON vs LLC) is denoted by *. Different lowercase letters denote an interaction effect considering Tukey’s adjusted *p* < 0.05. An *n* of 5–10 animals per group was used. Individual data point graphs for RT-PCR targets are available in Supplementary Fig. S3.

In males, differences in mean force production by grip strength did not reach statistical significance, even though a *p* = 0.07 was observed. In females mean force, a cancer main effect was noted (*p* < 0.05, Fig. 3a). Altered expression of calcium-specific channels and binding proteins are characteristic of lung cancer (Yang et al. 2010). Therefore, calcium-regulatory genes such as *Ryr1* and *Ser*ca1 were assessed in the plantaris and TA of both male and female mice. No differences on baseline expression of these genes were noted in male-WT-PBS vs female-WT-PBS (Supplementary Figs. S1a and S1b). In male plantaris, no differences in mRNA levels of *Ryr1* and *Serca1* were noted (Fig. 3b). Similarly, female mice did not show differences in mRNA levels of *Ryr1* and *Serca1* in both plantaris and TA muscles (Figs. 3c and 3e). Conversely, male TA muscle showed an interaction where WT-LLC showed double the mRNA content in *Ryr1* compared with PGC-PBS (*p* < 0.05, Fig. 3d).

### PGC1α overexpression differentially alters protein turnover-related genes and atrogenes expression in male and female mice

**Fig. 4.**
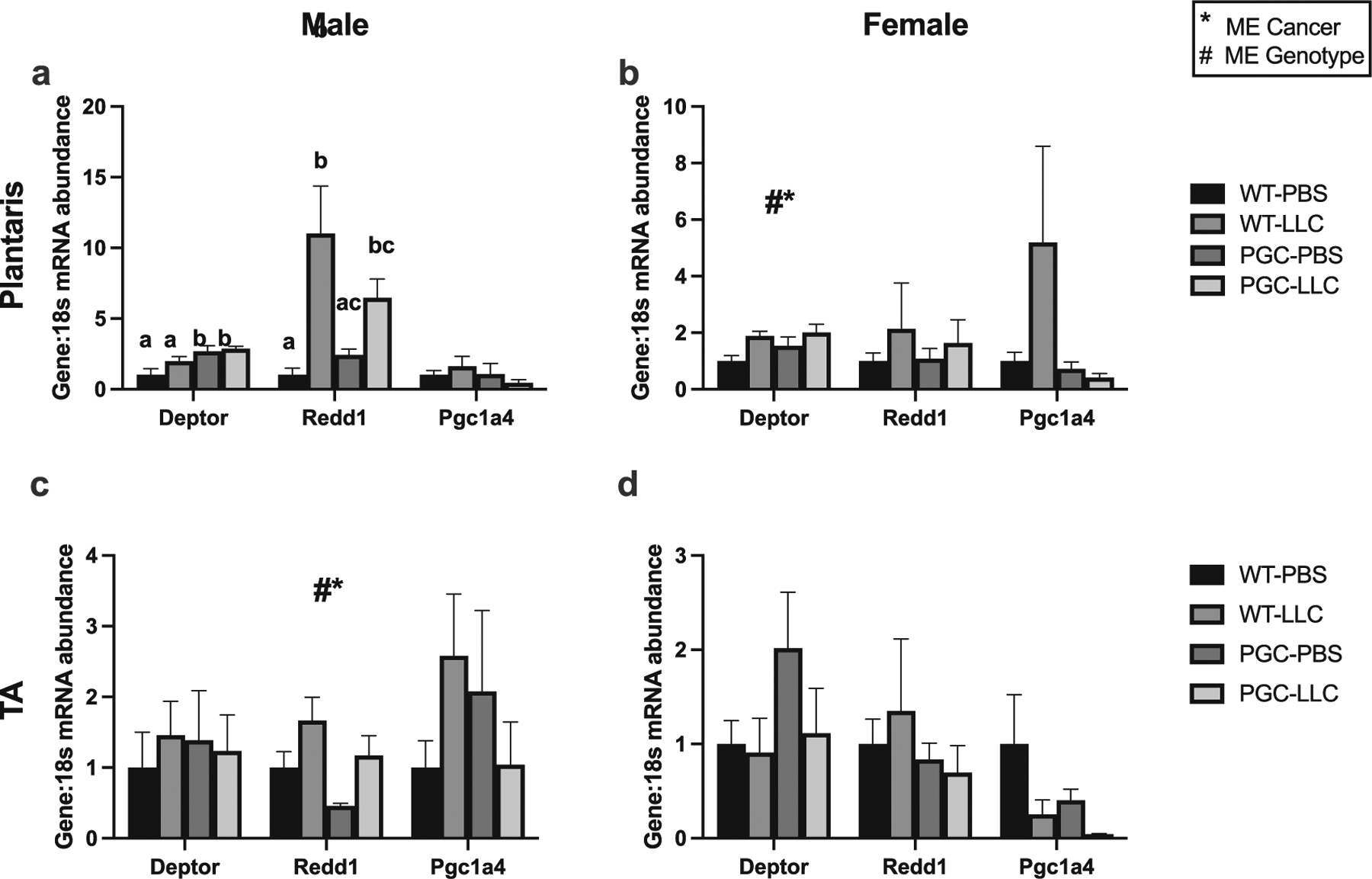
mRNA content of protein synthesis-associated genes in both plantaris and tibialis anterior muscles in males (*a*, *c*) and females (*b*, *d*), respectively. Data are shown in mean ± SEM. Main effect for cancer (CON vs LLC) is denoted by *. Main effect for genotype (WT vs PGC-1α) is denoted by #. Different superscript letters denote an interaction effect considering Tukey’s adjusted *p* < 0.05. An *n* of 5–10 animals per group was used. Individual data point graphs for RT-PCR targets are available in Supplementary Figs. S4a and S4b.

First, we have compared baseline expression of all RT-PCR targets of male-WT-PBS vs female-WT-PBS. No statistical differences were noted (Supplementary Fig. S1a and S1b). Then, to investigate if PGC1α transgenic overexpression mitigates alterations in protein turnover-related gene expression, we ran RT-PCR of *Redd1*, *Pgc1α4*, *Gadd45a*, *Atrogin*, *MuRF1*, and *Deptor*. In male plantaris, Deptor mRNA content displayed a genotype main effect, with PGC animals having 2.6-fold greater Deptor mRNA content compared with WT (p < 0.05, Fig. 4a). In female plantaris, both cancer and genotype main effects were noted, with PGC-LLC displaying ~3-fold greater in *Deptor* mRNA content compared with WT-PBS (*p* < 0.05, Fig. 4b). In male mice plantaris muscle, an interaction was noted for *Redd1* mRNA content with WT-LLC and PGC-LLC displaying, respectively, an ~11- and ~6-fold induction compared with WT-PBS, and WT-LLC being statistically different than PGC-PBS (*p* < 0.05, Fig. 4a). In females, plantaris *Redd1* mRNA levels did not reach statistical differences (Fig. 4b). In male TA muscle, no statistical difference was noted in *Deptor* (Fig. 4d). In addition, males TA muscle displayed both cancer and genotype main effect in *Redd1* mRNA content with LLC having ~35% greater mRNA content than PBS and WT displaying ~30% higher mRNA content than PGC (*p* < 0.05, Fig. 4c). In females, no significant changes in mRNA levels of *Redd1* were noted (Fig. 4d). In males TA and plantaris muscles, *Pgc1α4* mRNA content displayed no statistical differences (Fig. 4c). Similarly, in female mice both plantaris and TA muscle *Pgc1α4* mRNA content did not reach statistical difference, although a *p* = 0.08 was observed for cancer main effect in TA muscle (Figs. 4b and 4d).

**Fig. 5.**
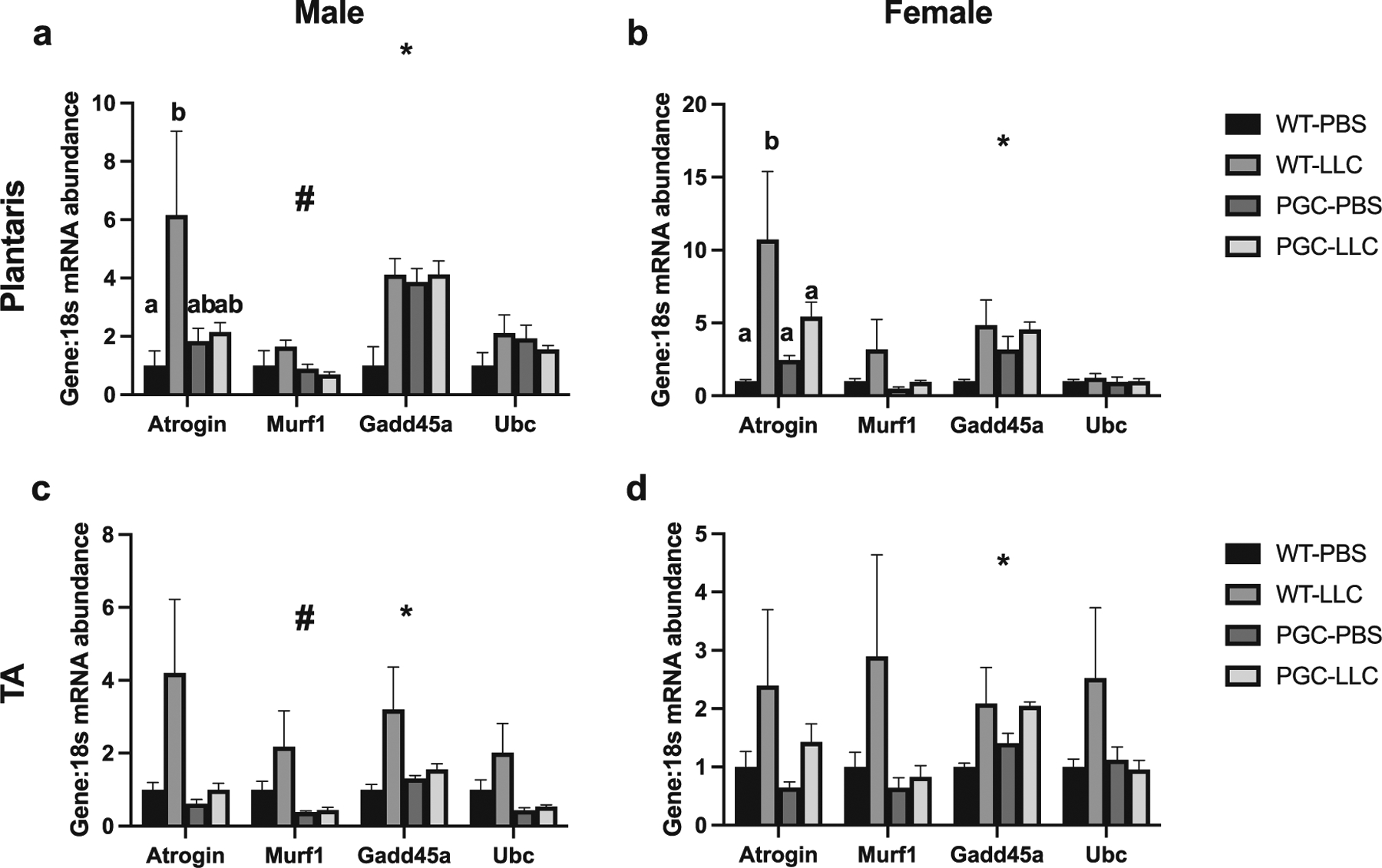
mRNA content of protein degradation-associated genes in both plantaris and tibialis anterior muscles in males (*a*, *c*) and females (*b*, *d*), respectively. Data are shown in mean ± SEM. Main effect for cancer (CON vs LLC) is denoted by *. Main effect for genotype (WT vs PGC-1α) is denoted by #. Different lowercase letters denote an interaction effect considering Tukey’s adjusted *p* < 0.05. An *n* of 5–10 animals per group was used. Individual data point graphs for RT-PCR targets are available in Supplementary Fig. S5a and S5b.

In male plantaris muscle, an interaction was noted in *Atrogin* mRNA content with WT-LLC displaying ~6-fold greater compared with WT-PBS (*p* < 0.05, Fig. 5a). Similarly, in female plantaris muscle, a cancer main effect was noted with LLC displaying ~7-fold greater *Atrogin* mRNA content compared with WT (*p* < 0.05, Fig. 5b). In male TA, no statistical difference in *Atrogin* mRNA content was noted, although *p* = 0.06 for genotype main effect and *p* = 0.07 for cancer main effect were noted (Fig. 5c). In females TA muscle, no statistical difference was seen, although *p* = 0.09 for cancer main effect was noted (Fig. 5d). In male plantaris muscle, *MuRF1* mRNA level did not reach statistical difference (Fig. 5a). In male TA, a genotype main effect was noted in *MuRF*1 mRNA content, with WT showing 2-fold greater *Murf1* mRNA content than PGC (*p* < 0.05, Fig. 5c). In females, both plantaris and TA muscle, there were no statistical differences in *MuRF1* mRNA content (Figs. 5b and 5d). In males plantaris muscle a cancer main effect was noted, where LLC group displaying ~1-fold more mRNA content of *Gadd45a* when compared with PBS (*p* < 0.05, Fig. 5a). In male TA muscle, a cancer main effect was observed in *Gadd45a* mRNA content (*p* < 0.05, Fig. 5c). In female mice both plantaris and TA muscle, a cancer main effect was noted in *Gadd45a* mRNA content, where LLC group displayed an ~1- and 2-fold greater mRNA levels of *Gadd45a* when compared with PBS, respectively (*p* < 0.05, Figs. 5b and 5d). In male mice TA muscle, a genotype main effect was noted in *Ubc* mRNA content, with PGC animals having ~50% lower *Ubc* content compared with WT (*p* < 0.05, Fig. 5c), while no differences in plantaris muscle (Fig. 5a). In females, both plantaris and TA muscle displayed no differences in *Ubc* mRNA (Figs. 5b and 5d).

### Mitochondrial reporter MitoTimer is differently changed by PGC-1α overexpression in male and female mice

**Fig. 6.**
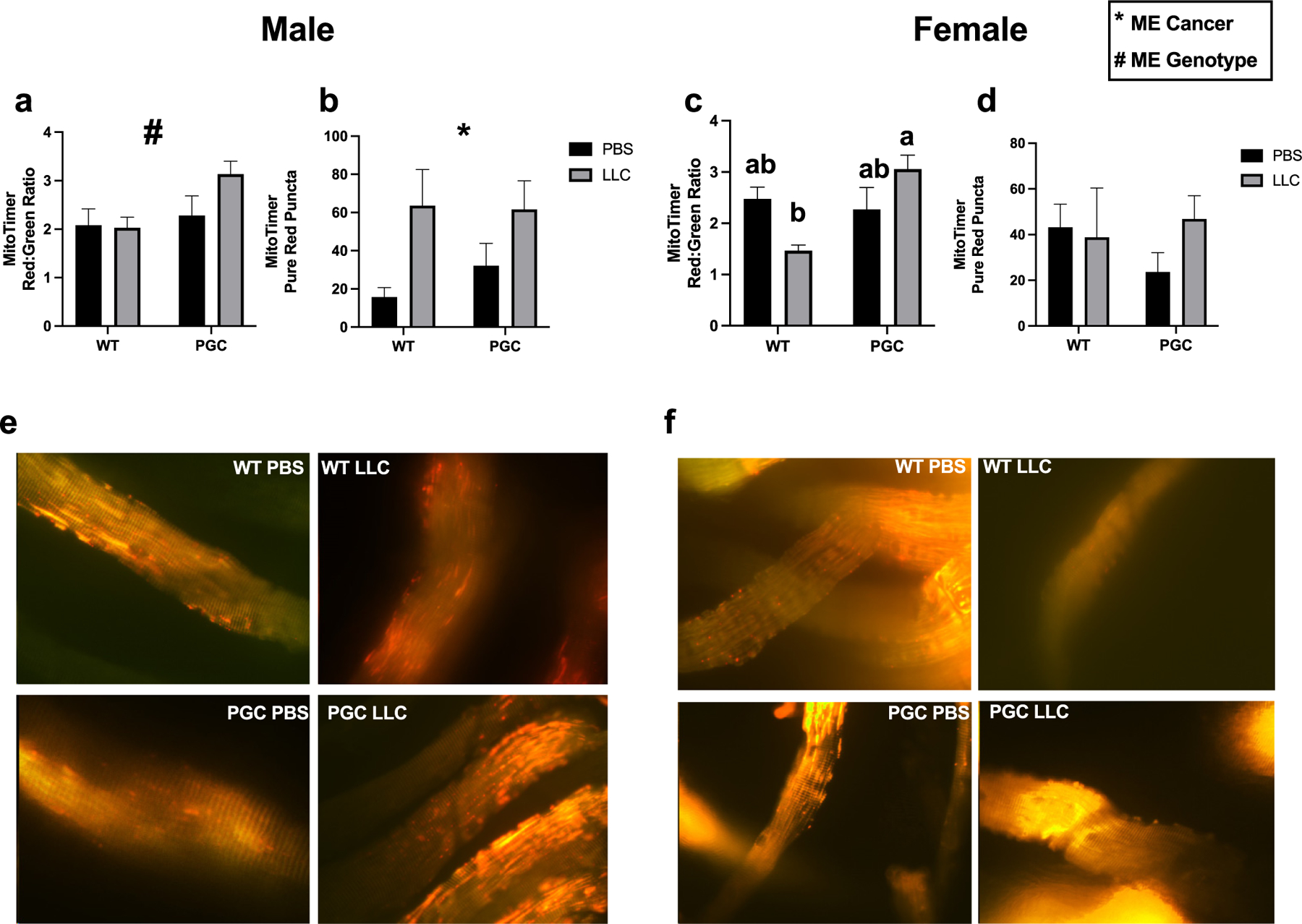
Mitochondria quality via MitoTimeR tracer in FDB muscle of both male and female mice. Red:green ratio quantification of both males (*a*) and females (*c*). Red puncta quantification of both males (*b*) and females (*d*). Representative images of MitoTimer were taken at 40× magnification and are shown for both males (*e*) and females (*f*) for all 4 conditions. Data are shown in mean ± SEM. Main effect for cancer (CON vs LLC) is denoted by *. Main effect for genotype (WT vs PGC-1α) is denoted by #.Different lowercase letters denote an interaction effect considering Tukey’s adjusted *p* < 0.05. An *n* of 8–10 animals per group was used.

Regarding MitoTimer, in males, a genotype main effect was observed in MitoTimer tracer of red:green ratio, with PGC showing ~32% higher red:green ratio than WT groups (*p* < 0.05, Figs. 6a and 6e). Alongside, a cancer main effect was noted in pure red puncta in males, where LLC displayed ~3-fold more red puncta than PBS groups (*p* < 0.05, Figs. 6b and 6e). In females, an interaction was noted in red:green ratio with WT-LLC displaying ~56% lower red:green when compared with PGC-LLC (*p* < 0.05, Figs. 6c and 6f). Females did not demonstrate significant changes in pure red puncta, where (*p* < 0.05, Figs. 6d and 6f).

### Pgc1α overexpression differently alters mitophagy-associated genes expression in male and female mice

**Fig. 7.**
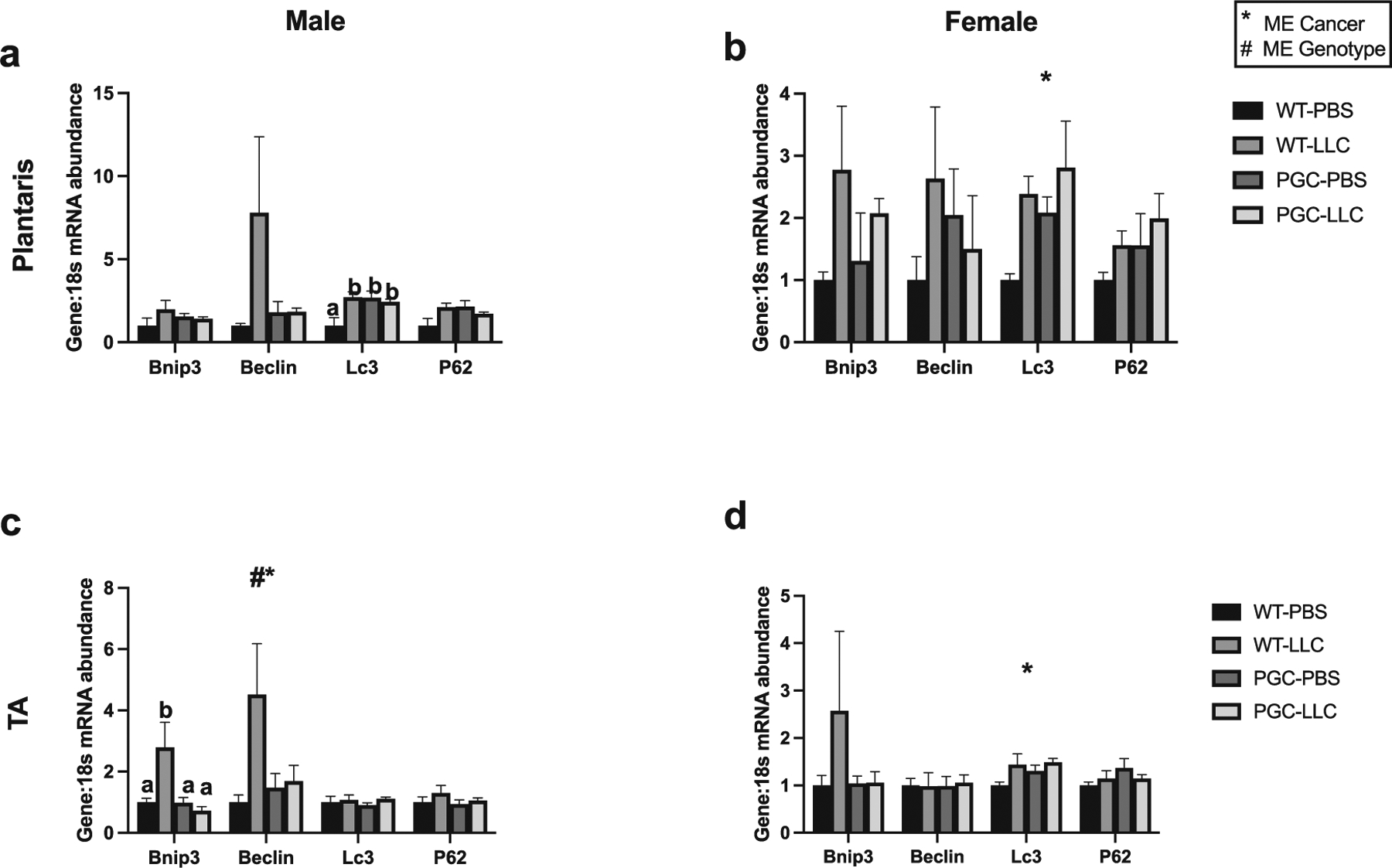
mRNA content of mitophagy-associated genes in both plantaris and tibialis anterior muscles in males (*a*, *c*) and females (*b*, *d*), respectively. Data are shown in mean ± SEM. Main effect for cancer (CON vs LLC) is denoted by *. Main effect for genotype (WT vs PGC-1α) is denoted by #. Different lowercase letters denote an interaction effect considering Tukey’s adjusted *p* < 0.05. An *n* of 5–10 animals per group was used. Individual data point graphs for RT-PCR targets are available in Supplementary Figs. S6a and S6b.

Firstly, we have compared baseline expression of all RT-PCR targets of male-WT-PBS vs female-WT-PBS. Then, to investigate in PGC1α transgenic overexpression mitigates impairments in genes related to mitophagy, we performed RT-PCR of *Bnip3*, *Beclin*, *Lc3*, and *p62*. In male plantaris muscle, no statistical difference was seen in *Bnip3* mRNA content (Fig. 7a). In male TA muscle, an interaction was noted with WT-LLC having ~2.8-fold greater *Bnip3* mRNA content compared with PGC-LLC and being significantly different from all the other groups (*p* < 0.05, Fig. 7c). In females, both plantaris and TA muscle *Bnip3* mRNA content did not reach statistical difference, although *p* value for cancer main effect was *p* = 0.07 in plantaris muscle (Figs. 7b and 7d). In male plantaris muscle, *Beclin* mRNA content showed no statistical difference, although *p* = 0.08 for cancer main effect (Fig. 7a). In male TA muscle, both cancer and genotype effects were noted for *Beclin* mRNA content, where WT-LLC displayed ~31% higher and PGC-PBS showed ~50% lower *Beclin* mRNA levels when compared with WT-PBS, and PGC-LLC had ~46% higher *Beclin* mRNA levels compared with PGC-PBS (*p* < 0.05, Fig. 7c). In females, both plantaris and TA muscle did not display statistical difference for *Beclin* mRNA content (Figs. 7b and 7d). In male plantaris muscle, an interaction was observed in *Lc3* mRNA content with WT-LLC and PGC-LLC displaying an ~2.7-and 2.5-fold greater mRNA content compared with WT-PBS and PGC-PBS having ~2.7-fold higher mRNA content compared with WT-PBS (*p* < 0.05, Fig. 7a). In female mice plantaris muscle, a cancer main effect was noted in *Lc3* with LLC displaying 1-fold induction compared with PBS (*p* < 0.05, Fig. 7b). No statistical difference was seen in *Lc3* mRNA content in TA muscle in male mice (Fig. 7c). In females TA muscle, a cancer main effect was noted for *Lc3* mRNA content, with LLC displaying ~30% more *Lc3* mRNA content than PBS groups (*p* < 0.05, Fig. 7d). In males plantaris muscle, an interaction was observed for *p62* mRNA content (*p* < 0.05, Fig. 7a). In male TA muscle, no statistical difference was seen in *p62* mRNA levels (Fig. 7c). In females, both plantaris and TA muscle showed no statistical difference in mRNA content of *p62* (Figs. 7b and 7d).

## Discussion

To our knowledge, this is one of few studies to investigate a mitochondrially targeted genetic intervention as potential prevention of cancer-associated muscle wasting in both biological sexes. In our cell culture experiment, MitoT ameliorated LCM-induced atrophy in vitro, which indicates mitochondria-targeted strategies could be viable for protection against CC. Considering PGC-1α as a coordinator of a multitude of mitochondrial genes, including antioxidants for protection against oxidative stress damage (Deng et al. 2020) we further explored mitochondrial potential for protection against CC by utilizing MCK-PGC1α transgenic animals. Although PGC overexpression may be protective in other forms of atrophy (Sandri et al. 2006; Cannavino et al. 2014, 2015; Wang et al. 2017), we found that *Pgc1α* overexpression is not sufficient to attenuate CC similar to prior works (Wang et al. 2012, 2017). However, similar to prior work from our laboratory displaying no protection by MCK-PGC1α in disuse atrophy (Rosa-Caldwell et al. 2020), our data suggest that while MCK-PGC1α attenuates the ubiquitin–proteasome response to atrophic stimuli in males TA muscle, this is likely due to a concomitant induction of autophagic protein degradation.

Recent studies from our laboratory have shown mitochondrial impairment, such as increased oxidative stress precede muscle wasting in cancer-induced atrophy with most robust effects in male mice (Brown et al. 2017b; Lim et al. 2022). Therefore, to determine the potential of mitochondria-based therapies in CC, we first utilized an in vitro experiment using the mitochondria-specific antioxidant MitoT in a model of LCM exposure-induced myotube atrophy. MitoT has shown positive outcomes in preventing cardiovascular dysfunction due to aging and myotube shrinkage derived from other sources of atrophy (Olgar et al. 2018; D.E. Lee et al. 2017; Mäkinen et al. 2017; Raiteri et al. 2021). Likewise, this study showed that MitoT was sufficient to preserve myotube diameter after 24 hours of LCM intervention which appears to be largely due to alleviation of LCM-stimulated protein degradation (protein ubiquitination immunoblot). However, it is important to highlight that due to female background of C2C12, these results should be carefully interpreted, as female muscle-derived stem cells are less sensitive to oxidative stress compared with male-derived (Deasy et al. 2007). These findings give the foundation to further exploration for targeting mitochondria to improve skeletal muscle impairments due to cancer. As PGC-1α plays a role in prevention of oxidative stress and mitochondrial impairments (Valle et al. 2005; Vazquez et al. 2013; Rius-Pérez et al. 2020), we then explored possible protection potential of PGC1*α*-overexpression by investigating effects of CC in MCK-PGC1α transgenic animals.

CC notoriously reduces muscle function and is characterized by a significant loss of muscle mass. Prior studies using MCK-PGC1α transgenic animals show attenuation of protein degradation pathways, including studies showing that these alterations were sufficient to promote phenotypic improvements against cachexia (Sandri et al. 2006; Sacheck et al. 2007; Brault et al. 2010; Cannavino et al. 2014, 2015; Kang and Ji 2016; Wang et al. 2017). Contrary to some of these findings, we did not note protection from muscle wasting, nor protection of muscle force production in the present study. In summary, both female and male mice exhibited decreased wet muscle weight of most measured hindlimb muscles such as gastrocnemius, extensor digitorum longus (EDL), and TA due to cancer. This suggests reduction in muscle mass in both male and female mice across different MHC Type II predominant fiber type muscles. It is important to highlight that there are major differences in muscle wasting models and distinct responses are expected as far as protection from muscle wasting via *Pgc1α*. For instance, some of these studies used an aging model in 24-week-old mice (Cannavino et al. 2014, 2015) or denervation-induced atrophy (Sandri et al. 2006), while others used a disuse model of muscle atrophy (Rosa-Caldwell et al. 2020). Different models of muscle wasting work through varying mechanisms. For instance, CC displays a much more pronounced muscle wasting derived by inflammation in contrast to aging or disuse-induced muscle pathologies. Lack of protection was also noted in muscle force in this study. Even though not significant in male mice, mean force normalized by body weight displayed a pattern where all LLC groups had lower force outcomes. In females, a similar pattern was noted more prominently, where a cancer main effect was observed, suggesting in this study female mice could display more robust losses in voluntary muscle force earlier than male mice in LLC-induced cachexia. We also speculate that loss of force was dependent on muscle mass loss along with preferential protection of slow twitch fibers in early stages of cachexia, that goes with prior findings from our group of preservation of oxidative fibers in male tumor-bearing mice (Brown et al. 2017b).

Cancer-induced muscle wasting is the result of an unbalanced rate of protein turnover whereby protein breakdown exceeds protein synthesis. Regarding the regulation of muscle protein synthesis, male mice displayed greater induction of anabolic/mTORC1 repressors *Deptor* and *Redd1* across TA and plantaris muscles compared with female mice. *Pgc1α* overexpression appeared to increase *Deptor* mRNA levels in male plantaris muscle, while in females, cancer and genotype seemed to induce an increase in *Deptor* mRNA levels in plantaris. Regarding *Redd1*, induction was noted in both plantaris and TA in male mice, while females remained unchanged. We previously observed changes in protein and mRNA levels of *Deptor* and *Redd1* in both male and female LLC tumorbearing mice (Brown et al. 2018; Lim et al. 2022), suggesting inductions of these anabolic repressors may be responsible for noted impairments in protein synthesis in tumor-bearing mice (White et al. 2012; Brown et al. 2018; Lim et al. 2022).

Conversely, regarding protein degradation, signaling cascades through ubiquitin and E3 ligases including Ubc, Atrogin, and MuRF1 play an important role on weighing this scale towards protein degradation, resulting in muscle wasting. Like prior studies, we found *Pgc1α* was sufficient to blunt induction of some of the key regulators of protein degradation such as Atrogin and MuRF1 (Sandri et al. 2006; Rosa-Caldwell et al. 2020) despite a lack of protection to muscle mass. Even though the mechanism behind atrophy stimuli are inherently different, we found similar results in attenuation in *Atrogin* and *Murf1* mRNA content in similar patterns in male and females without protection in phenotypic outcomes (i.e., muscle size and grip strength). Furthermore, although not significant in females, we noted a pattern of blunted mRNA content for *Ubc* in plantaris muscle. Meanwhile, this blunting effect of *Pgc1α* overexpression on *Ubc* mRNA content was significant in male TA muscle. This result is similar to prior reports (Rosa-Caldwell et al. 2020), and further confirms that changes in classical cellular signaling are not necessarily sufficient to blunt loss of muscle mass nor as markers to denote the occurrence of muscle loss itself, thus, interventions that rely only on cellular signaling manipulation should consider evidence like this for future investigation.

Even though phenotype remained unchanged by *Pgc1α* overexpression, our data agree with prior reports utilizing the MitoTimer reporter and mRNA markers for insight into mitochondrial quality and its regulation. Our MitoTimer data suggest *Pgc1α* overexpression is not sufficient to mitigate mitochondrial damage and a disrupted network. Degenerated mitochondria represented by red puncta in Fig. 3 was elevated in males by 4-fold in both cancer groups compared with WT PBS with a statistical cancer effect. In females there was a statistical cancer effect with no noticeable changes in red puncta for PGC groups. Interestingly, red:green ratio was higher in PGC groups and even more in PGC-LLC by ~1-fold and ~2-fold in males and females, respectively. Moreover, basal mitophagy seems to be elevated due to *Pgc1α* overexpression. Lira et al. (Lira et al. 2013) showed an increase in protein content of BNIP3, and LC3 with exercise intervention derived by elevated *Pgc1α*. Accordingly, recent data from our laboratory showed elevated LC3 protein content in both male and female PGC transgenic mice under hindlimb suspension (Rosa-Caldwell et al. 2020). In the present study, we found elevated mRNA content of *Lc3* in all PGC groups and LLC in both male and female mice. Therefore, blunted induction of the ubiquitin–proteasome pathway previously discussed here and previously noted by our group during disuse-induced atrophy in PGC transgenic mice is likely due to a greater reliance upon autophagic pathways of protein degradation following *Pgc1α* overexpression (Rosa-Caldwell et al. 2020). Interestingly, elevations in *Bnip3* mRNA content as a result of LLC tumor seemed to be attenuated with *Pgc1α* overexpression in TA muscle in male, and a similar pattern was noted with female mice and *Beclin* mRNA content in both plantaris and TA in male mice. Male mice seem to display more prominent changes in reduction of mitophagy markers, and this could be the result of lesser mitochondrial efficiency or “basal protection” of mitochondria in males compared with females. Recent studies from our group and others support this hypothesis, whereby female mice seem to display blunted oxidative stress through induction of autophagy and mitophagy compared with male mice submitted to hindlimb unloading (Brown et al. 2017b; Rosa-Caldwell et al. 2020, 2021; Lim et al. 2022). We hypothesize that although mitochondrial biogenesis is elevated, it is not sufficient to improve mitochondrial quality and prevent muscle loss induced by cancer, thus, does not protect from muscle wasting and reduce mRNA content of important mitophagy promoters such as *Bnip3* and *Beclin* possibly reducing clearance of damaged mitochondria noted by increased red puncta.

In conclusion, utilizing treatment with the mitochondriatargeted antioxidant MitoT in vitro we demonstrate that mitochondria-targeted therapies exhibit significant potential in the treatment and prevention of cancer-induced cachexia. Subsequently, we utilized in vivo *Pgc1α* overexpression via muscle-specific transgenic mice to determine if targeting PGC-1α to broadly promote mitochondrial quality control systems exhibits efficacy to prevent CC. We find *Pgc1α* overexpression is sufficient to blunt key protein degradation pathways yet is not sufficient to protect against cancer-induced muscle wasting. Similar to our prior work in disuse-induced atrophy it appears that during *Pgc1α* overexpression reliance of protein degradation is instead shifted toward autophagic systems. This shift demonstrates the insufficiency of utilizing classic degradative markers to interpret impacts on muscle atrophy. *Pgc1α* overexpression did not protect mitochondrial health during cancer-induced cachexia suggesting broad promotion of quality control systems is not capable of providing protection from cachexia via promotion of mitochondrial health. Although *Pgc1α* overexpression did not protect mitochondrial health or muscle mass during cancer-induced cachexia, our in vitro data suggest the potential viability of mitochondria targeted therapeutics and suggest the need to further explore potential approaches therein.

## Supplementary Material

Data supplement

## Figures and Tables

**Table 1a.** Characteristics of the female mice.

	WT PBS	WT LLC	PGC PBS	PGC LLC	Main effect
Body weight (g)	20.5 ± 0.2	21.2 ± 0.4	20.9 ± 0.3	22.1 ± 0.7	
Tumor weight (mg)	N/A	2315.5 ± 562.3	N/A	2521.8 ± 546.6	CANCER
Body weight-tumor weight (mg)	20.5 ± 0.2	19.1 ± 0.3	20.9 ± 0.3	19.6 ± 0.3	CANCER
Gastrocnemius (mg)	105.7 ± 1	91.7 ± 2.7	104.9 ± 2.1	96 ± 1.9	CANCER
Soleus (mg)	9.1 ± 0.6	7.7 ± 0.6	10.1 ± 0.7	9.2 ± 0.4	GENOTYPE
Plantaris (mg)	14.7 ± 0.5	13.8 ± 0.6	16.2 ± 0.6	14.6 ± 0.5	CANCER + GENOTYPE
EDL (mg)	8.6 ± 0.5	7.9 ± 0.4	9.5 ± 0.5	8.6 ± 0.6	
TA (mg)	40.4 ± 0.7	35.6 ± 1.4	44.7 ± 1.1	40.3 ± 1.2	CANCER + GENOTYPE
Fat (mg)	209.1 ± 16.5	101.9 ± 13.3	208.6 ± 22.4	146.5 ± 17.3	CANCER
Heart (mg)	111.3 ± 4	104.7 ± 2.1	106.8 ± 13.4	114 ± 4.6	
Liver (mg)	966.9 ± 43.8	1086.8 ± 42.1	936.3 ± 67.2	1148 ± 51.9	CANCER
Spleen (mg)	83.2 ± 4	250.4 ± 40.1	90.2 ± 3.9	571.1 ± 214.1	CANCER

**Note:** All values are represented as means ± SEM. Female mice tissue weights are recorded as wet weights. An *n* of 8–10 animals per group was used. EDL, extensor digitorum longus; N/A, not applicable.

**Table 1b.** Characteristics of the male mice.

	WT PBS	WT LLC	PGC PBS	PGC LLC	Main effect
Body weight (g)	25.8 ± 0.6	27.9 ± 0.7	23.5 ± 0.7	27.2 ± 0.7	
Tumor weight (mg)	N/A	2118 ± 341.4	N/A	2787.8 ± 548.1	CANCER
Body weight-tumor weight (mg)	25.8 ± 0.6	25.1 ± 0.7	23.5 ± 0.7	25.1 ± 0.6	
Gastrocnemius (mg)	141 ± 4.2	129.7 ± 7.7	148.5 ± 5.1	135.2 ± 3.1	CANCER
Soleus (mg)	11.3 ± 0.5	10.4 ± 0.9	12.8 ± 0.6	11.8 ± 0.3	GENOTYPE
Plantaris (mg)	21.1 ± 0.8	18.3 ± 1.2	21.7 ± 0.8	20.8 ± 0.6	
EDL (mg)	11.8 ± 0.5	10.6 ± 0.8	12.4 ± 0.8	11 ± 0.3	CANCER
TA (mg)	53.3 ± 0.5	49.7 ± 3.3	58.2 ± 1.5	52.9 ± 1.2	CANCER
Fat (mg)	540.4 ± 33.2	265.3 ± 35.7	494.8 ± 25.4	330.4 ± 23.3	CANCER
Heart (mg)	133.8 ± 4	137.6 ± 6	139.2 ± 6.2	135.5 ± 2.8	CANCER
Liver (mg)	1172.4 ± 35.5	1181.7 ± 155.9	1241.3 ± 80.3	1394.7 ± 47.1	
Spleen (mg)	89.4 ± 5.6^A^	379.8 ± 39.5^B^	94.7 ± 6.1^A^	240.8 ± 27.7^C^	INTERACTION

**Note:** All values are represented as means ± SEM. Male mice tissue weights are recorded as wet weights. Different superscript letters denote an interaction effect considering Tukey’s adjusted *p* < 0.05. An *n* of 8–10 animals per group was used. EDL, extensor digitorum longus; N/A, not applicable.

## Data Availability

Data available upon request to the corresponding authors.
