# Supplementary material for: PGC-1α overexpression is not sufficient to mitigate cancer cachexia in either male or female mice
Source: Appl Physiol Nutr Metab. Author manuscript; Available in PMC 2023 May 22. (PMC10201462; doi:10.1139/apnm-2022-0086)
Supplement: Data supplement [file NIHMS1900024-supplement-Data_supplement.pdf]

Supplementary Figure 1a

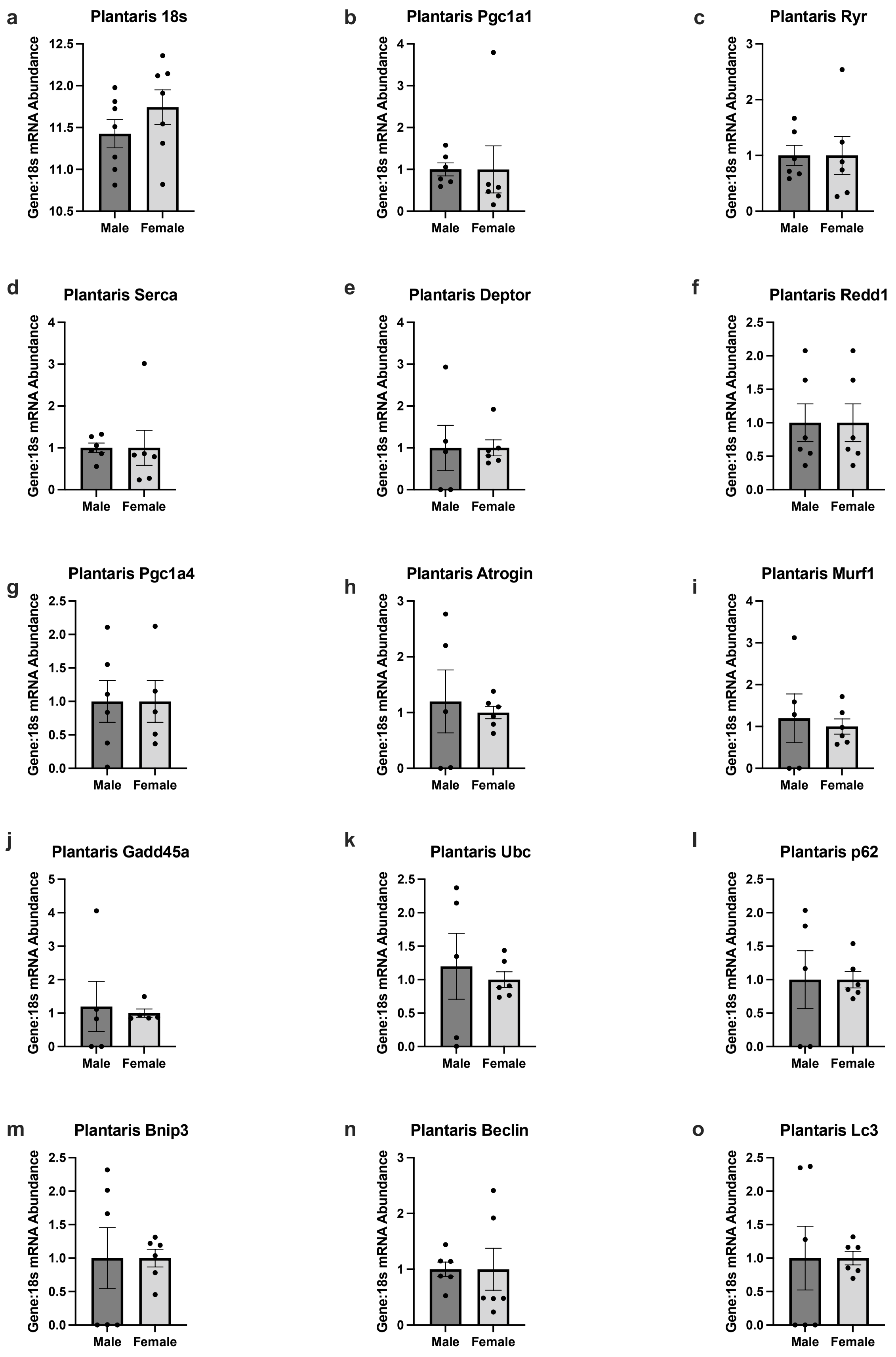

Supplementary Figure 1b

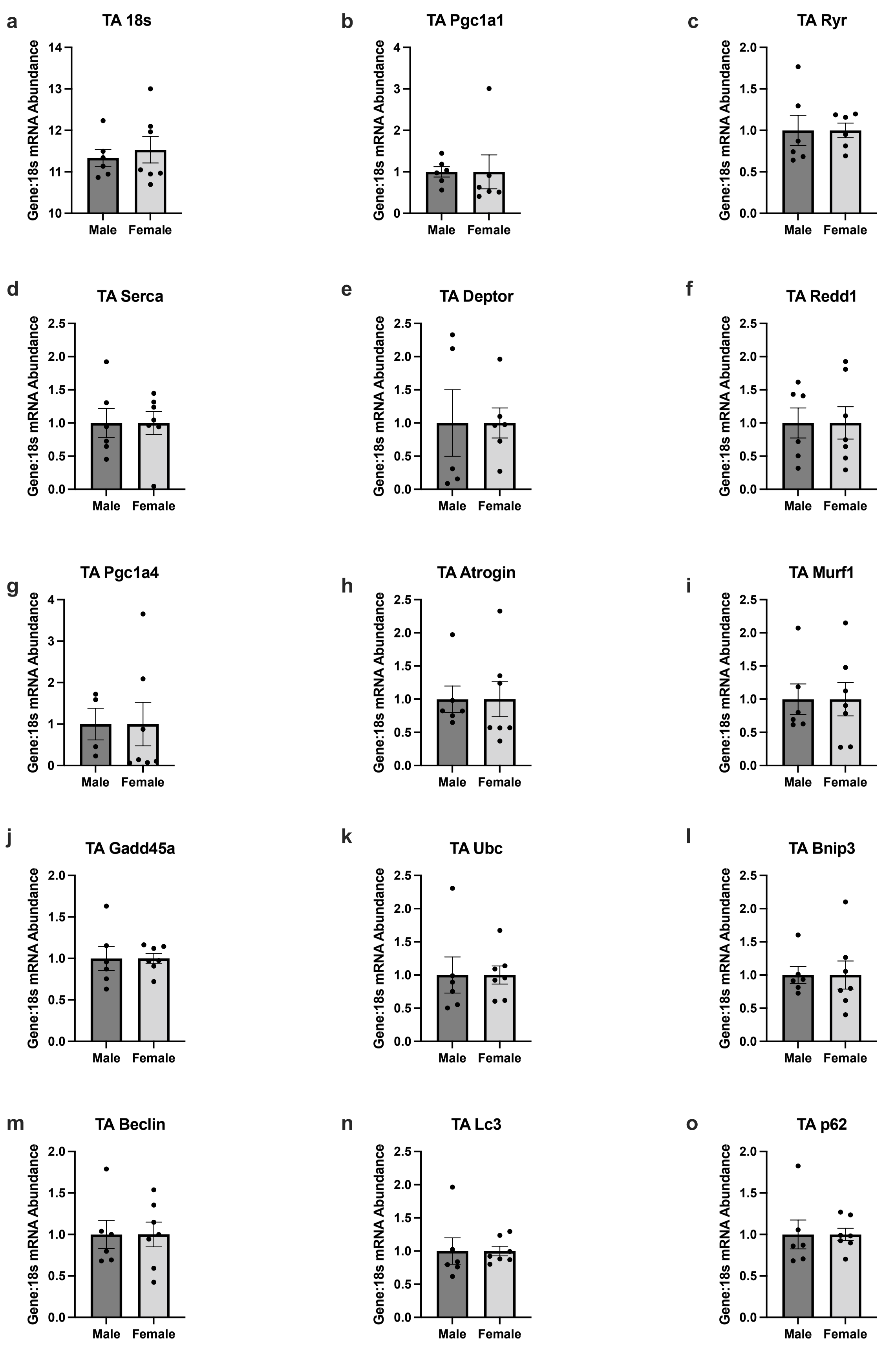

Supplementary Figure 2

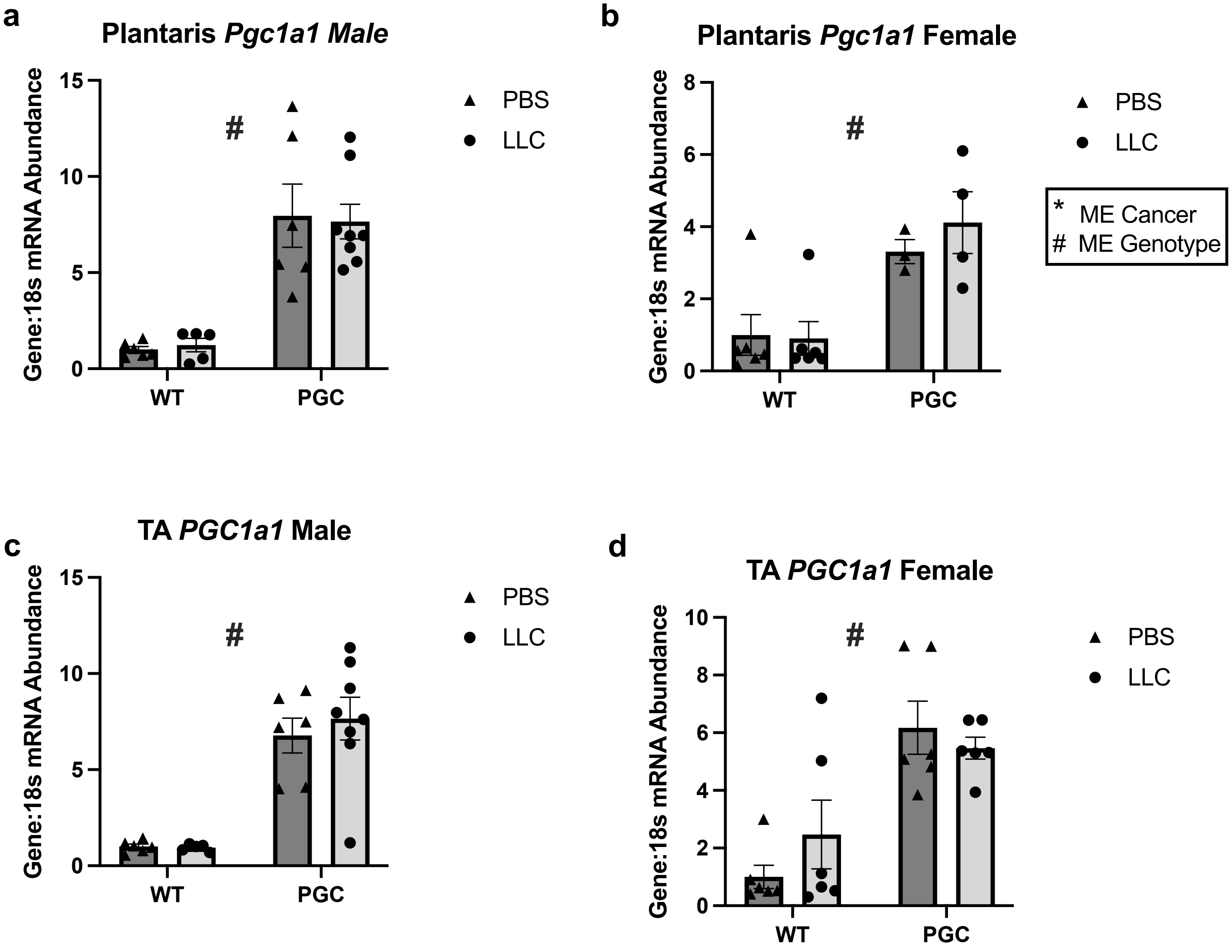

Supplementary Figure 3

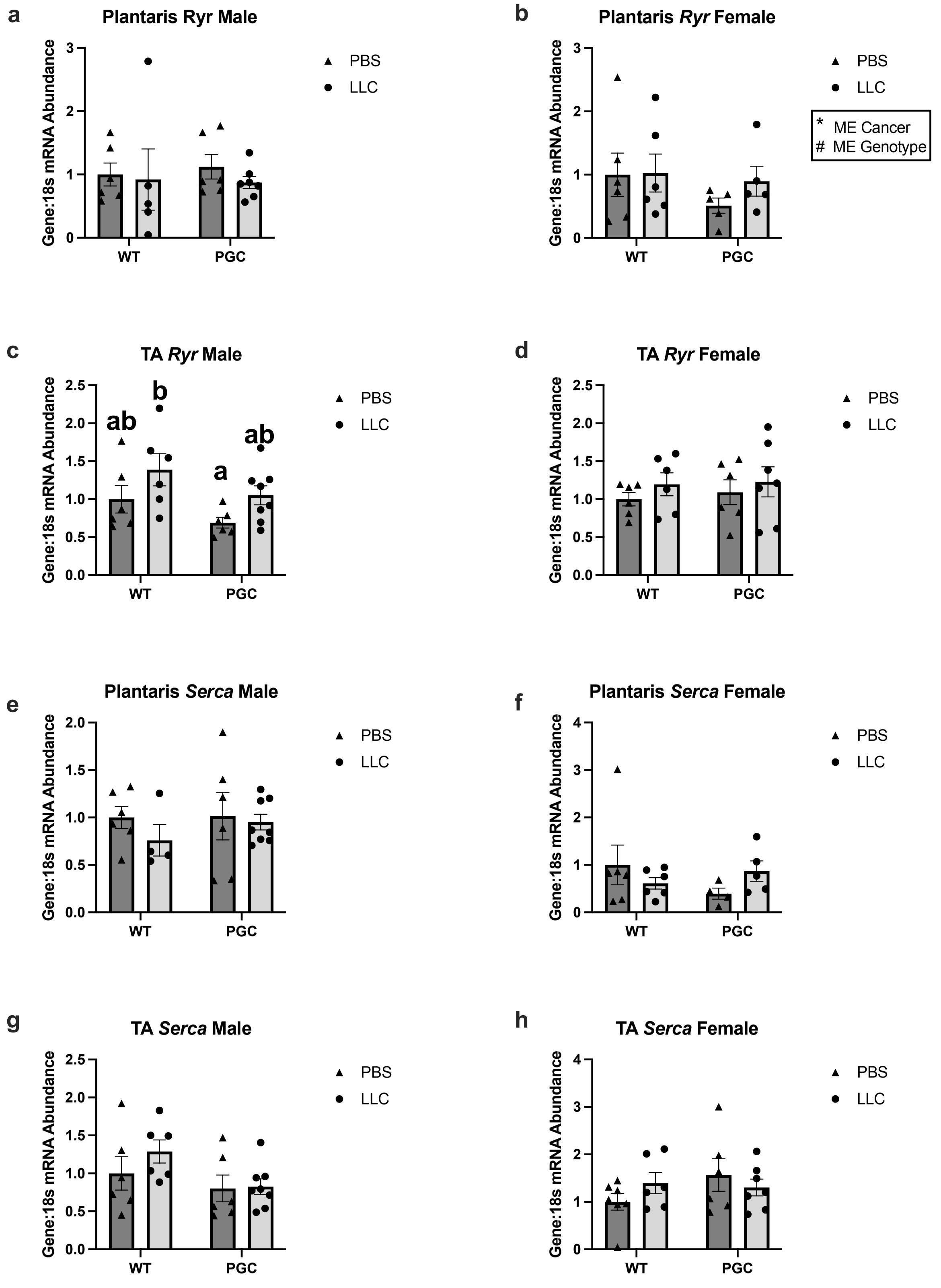

Supplementary Figure 4a

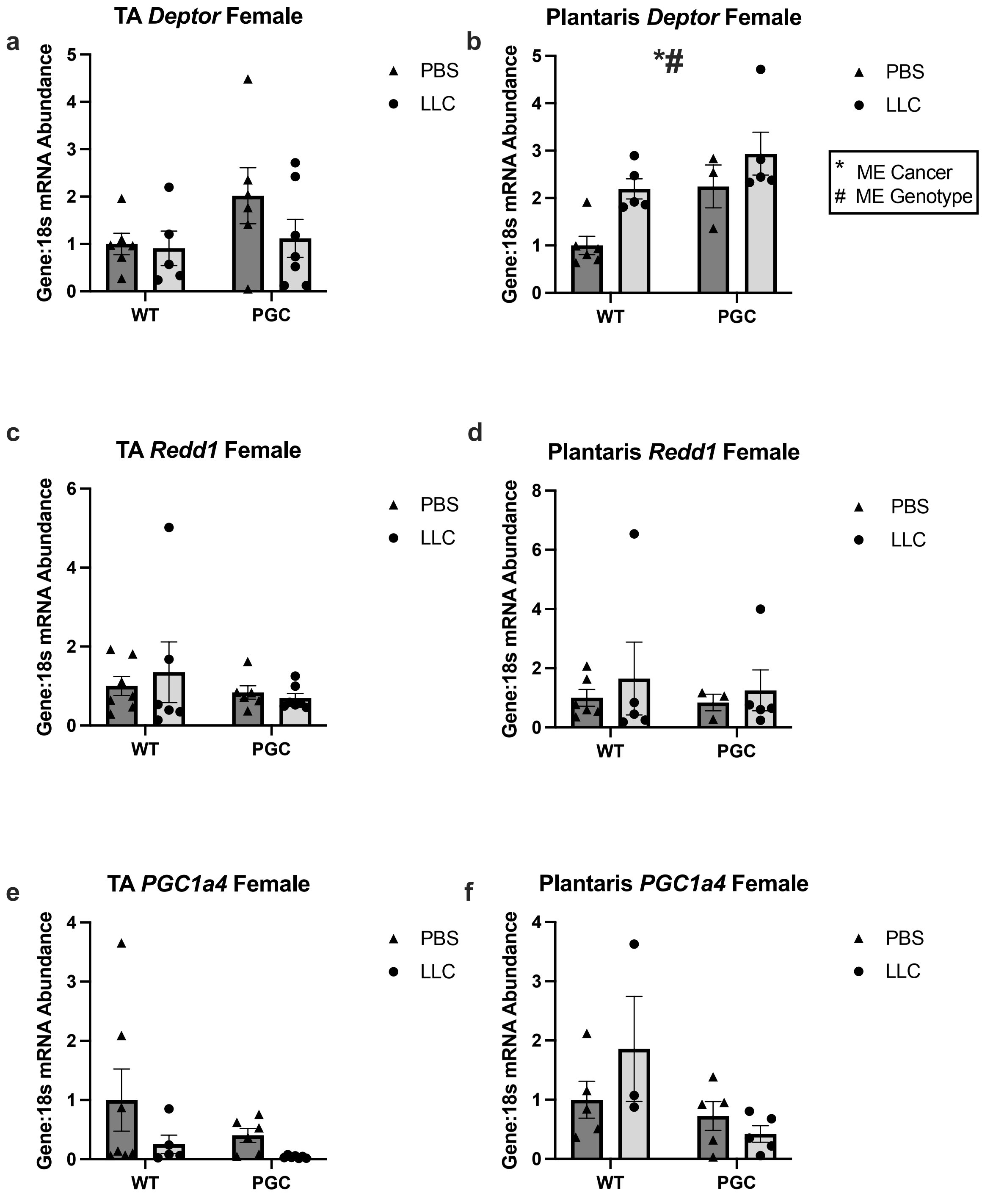

Supplementary Figure 4b

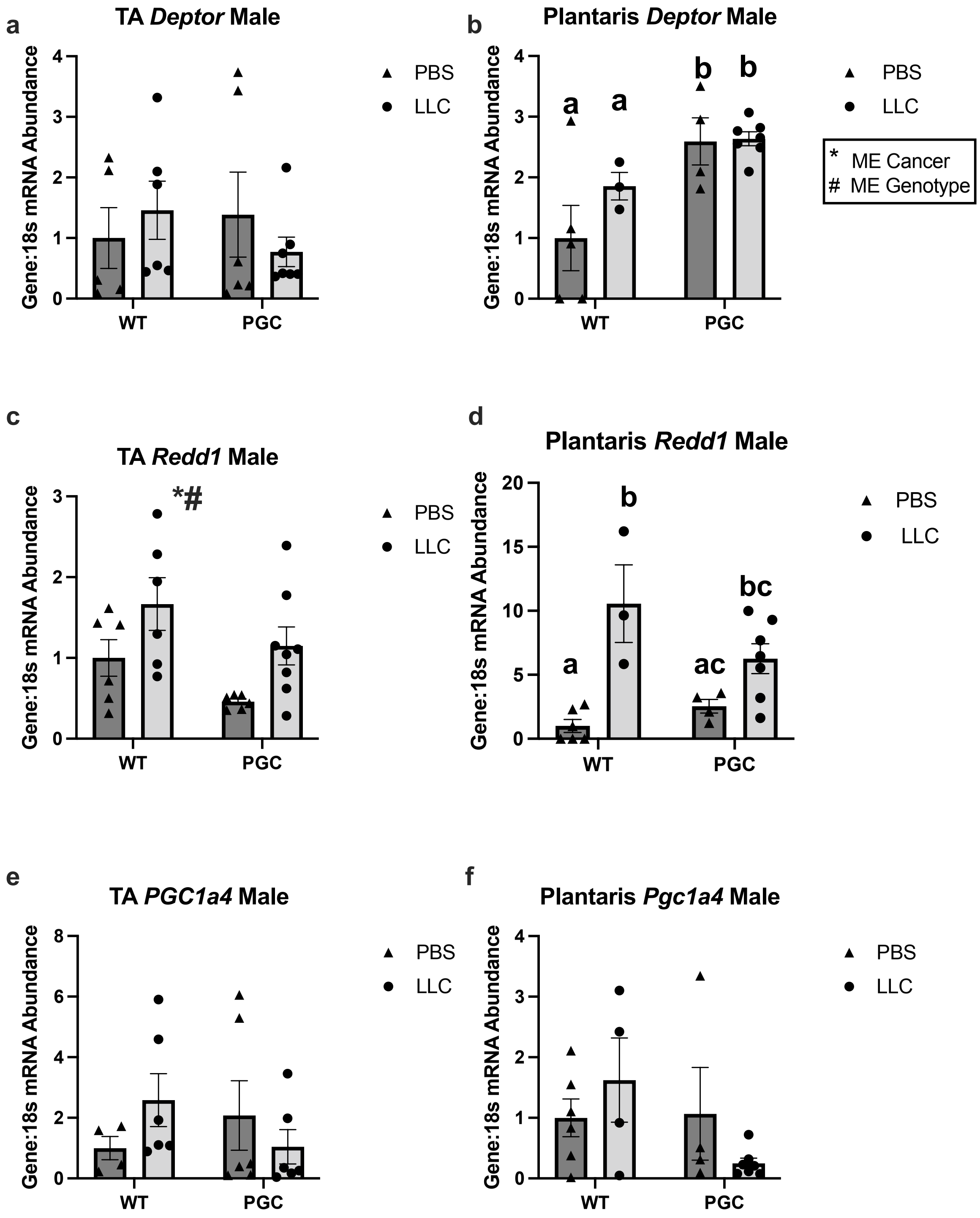

Supplementary Figure 5a

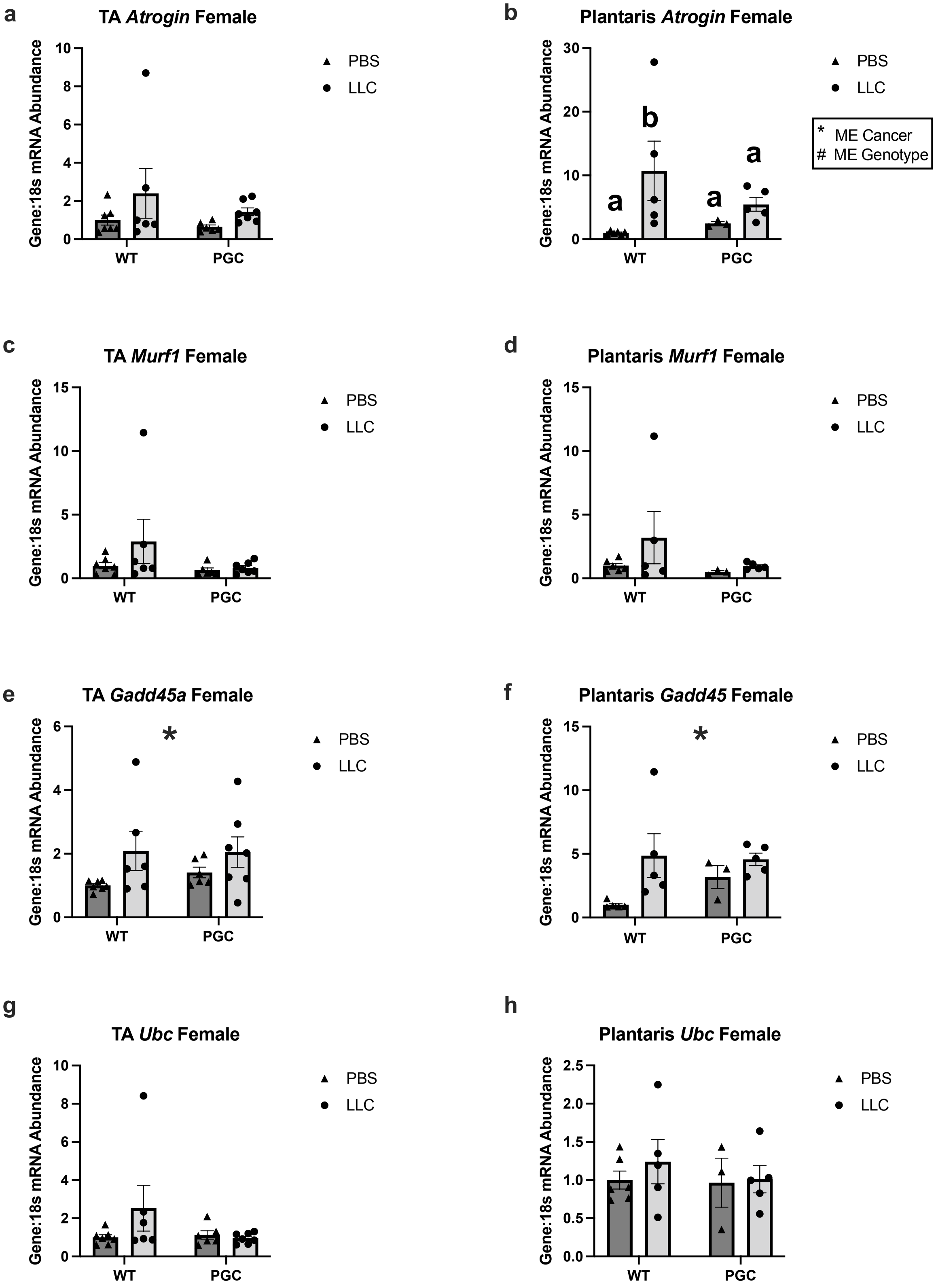

Supplementary Figure 5b

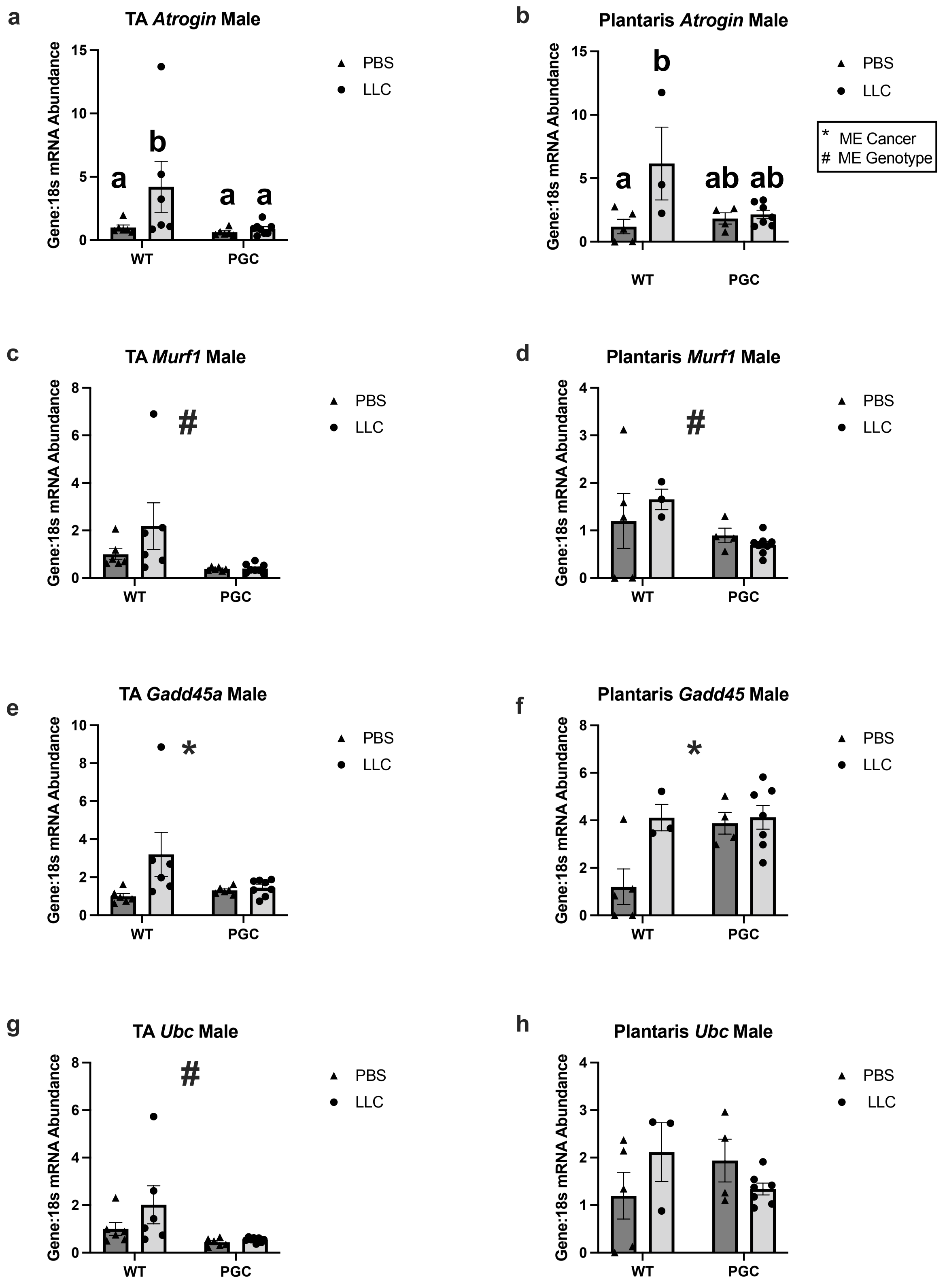

Supplementary Figure 6a

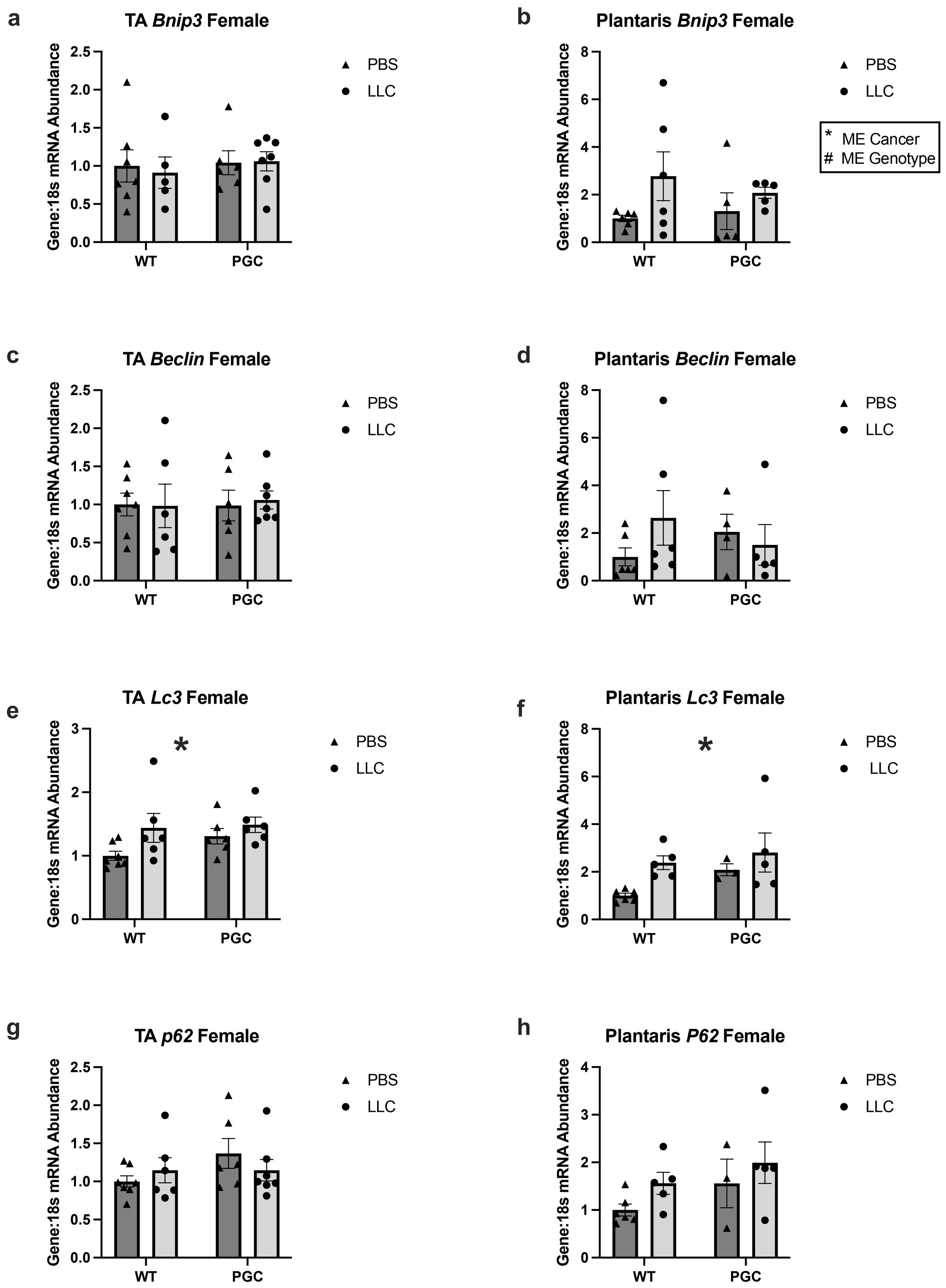

Supplementary Figure 6b

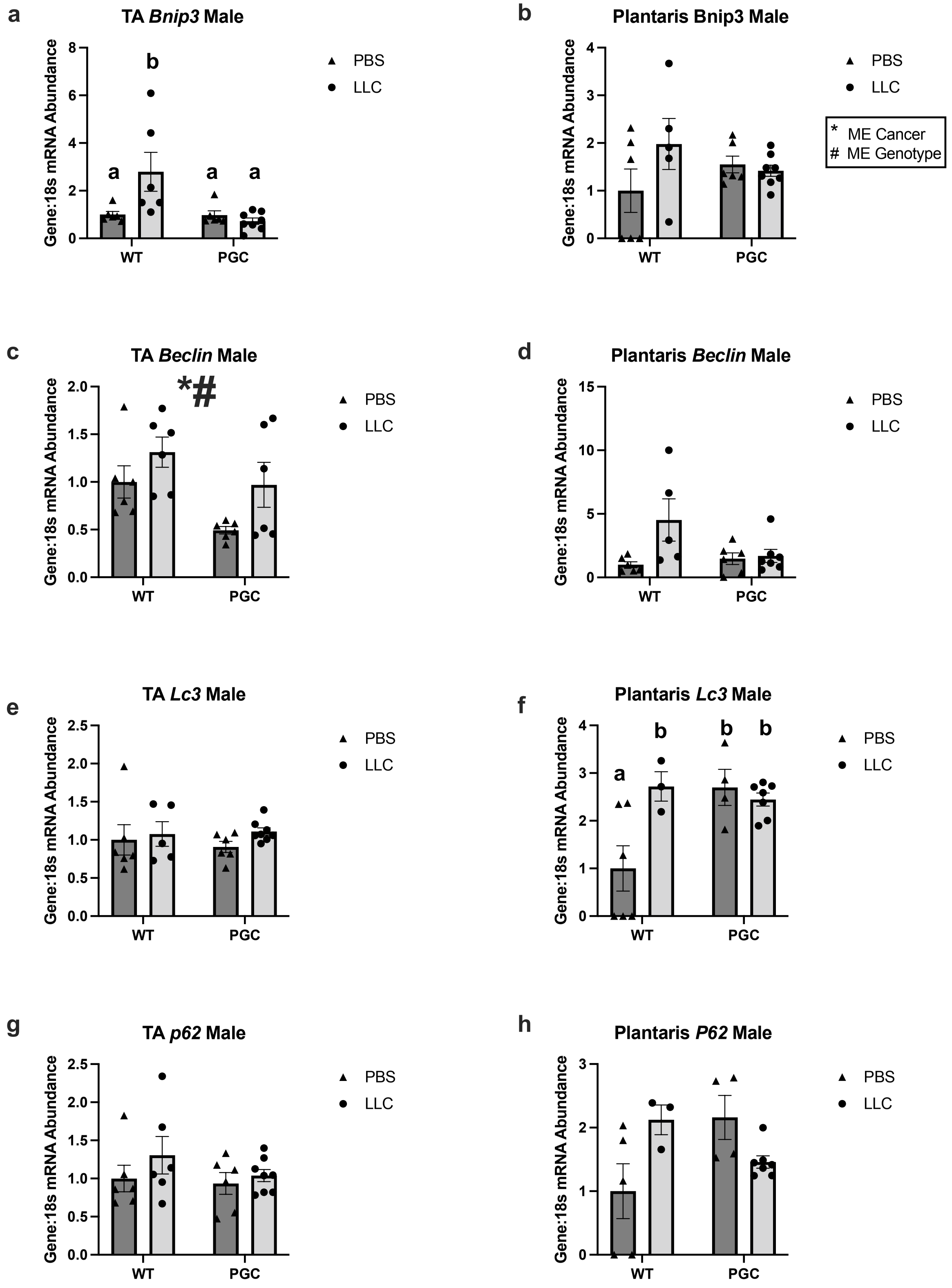

Figure Legends

Supplementary Figure 1a. Baseline comparison of Male WT-PBS vs Female WT-PBS groups, for all q-PCR targets of plantaris muscle via unpaired T-test, $p<0.05$.

Supplementary Figure 1b. Baseline comparison of Male WT-PBS vs Female WT-PBS groups, for all q-PCR targets of TA muscle via unpaired T-test, $p<0.05$.

Supplementary Figure 2. Individual data set for PGC1-α overexpression genotype confirmation. Data are shown in mean ± SEM. Main Effect for cancer (CON vs LLC) is denotated by *. Main Effect for genotype (WT vs PGC1-**α**) is denotated by **#,** $p<0.05$. A n of 5-10 animals per group was used.

Supplementary Figure 3. Individual data set for calcium-modulation associated genes. Data are shown in mean ± SEM. Main Effect for cancer (CON vs LLC) is denotated by *. Main Effect for genotype (WT vs PGC1-**α**) is denotated by **#,** $p<0.05$. A n of 5-10 animals per group was used.

Supplementary Figure 4a. Individual data set for protein synthesis associated genes in both Tibialis anterior, and Plantaris muscles in females. Data are shown in mean ± SEM. Main Effect for cancer (CON vs LLC) is denotated by *. Main Effect for genotype (WT vs PGC1-**α**) is denotated by **#,** $p<0.05$. A n of 5-10 animals per group was used.

Supplementary Figure 4b. Individual data set for protein synthesis associated genes in both Tibialis anterior, and Plantaris muscles in males. Data are shown in mean ± SEM. Main Effect for cancer (CON vs LLC) is denotated by *. Main Effect for genotype (WT vs PGC1-**α**) is denotated by **#,** $p<0.05$. A n of 5-10 animals per group was used.

Supplementary Figure 5a. Individual data set for protein degradation associated genes in both Plantaris and Tibialis anterior muscles in females. Data are shown in mean ± SEM. Main Effect for cancer (CON vs LLC) is denotated by *. Main Effect for genotype (WT vs PGC1-**α**) is denotated by **#,** $p<0.05$. A n of 5-10 animals per group was used.

Supplementary Figure 5b. Individual data set for protein degradation associated genes in both Tibialis anterior, and Plantaris muscles in males. Data are shown in mean ± SEM. Main Effect for

cancer (CON vs LLC) is denotated by *. Main Effect for genotype (WT vs PGC1-**α**) is denotated by **#,** $p<0.05$. A n of 5-10 animals per group was used.

Supplementary Figure 6a. Individual data set for mitophagy associated genes in both Plantaris and Tibialis anterior muscles in females. Data are shown in mean ± SEM. Main Effect for cancer (CON vs LLC) is denotated by *. Main Effect for genotype (WT vs PGC1-**α**) is denotated by **#,** $p<0.05$. A n of 5-10 animals per group was used.

Supplementary Figure 6b. Individual data set for mitophagy associated genes in both Tibialis anterior, and Plantaris muscles in males. Data are shown in mean ± SEM. Main Effect for cancer (CON vs LLC) is denotated by *. Main Effect for genotype (WT vs PGC1-**α**) is denotated by **#,** $p<0.05$. A n of 5-10 animals per group was used.
